# HIIT-induced lactate/GPR81 signaling with dual branches converging on ERK1/2 contributes to hippocampal synaptic remodeling and memory improvement

**DOI:** 10.3389/fcell.2026.1699042

**Published:** 2026-01-14

**Authors:** Xuepeng Bian, Qinghui Shang, Lutao Zhu, Jun Liu, Min Wu, Jingjing Li, Shujie Lou

**Affiliations:** 1 Department of Rehabilitation, School of International Medical Technology, Shanghai Sanda University, Shanghai, China; 2 School of Exercise and Health, Shanghai University of Sport, Shanghai, China; 3 Physical Education College, Shanghai University, Shanghai, China

**Keywords:** ERK1/2, GPR81, HIIT, hippocampus, memory, synaptic remodeling

## Abstract

The remodeling of synapses in the hippocampus is intricately linked to processes of learning and memory. Research indicates that high-intensity interval training (HIIT) enhances cognitive functions reliant on the hippocampus in mice, although the specific receptor-mediated molecular pathways involved are not fully elucidated. Lactate, which is produced in significant amounts during HIIT, may function as a signaling agent in the brain through the lactate receptor G protein-coupled receptor 81 (GPR81), classified as a Gi-type G protein-coupled receptor. This investigation focused on the lactate/GPR81 pathway’s contribution to synaptic remodeling in the hippocampus induced by HIIT and examined its downstream signaling characteristics. *In vivo* results demonstrated that HIIT led to an increase in dendritic spine density and presynaptic vesicle density, enhancing learning and memory; however, these structural and cognitive improvements were negated by the knockdown of GPR81 in the hippocampus. *In vitro* experiments with Neuro-2a (N2a) cells, when treated with a GPR81 agonist alongside an adenyl cyclase (AC) agonist, a phospholipase C (PLC) inhibitor, and an extracellular signal-regulated kinase 1/2 (ERK1/2) inhibitor, revealed that GPR81 activation resulted in elevated ERK1/2 phosphorylation and increased levels of proteins associated with synaptic remodeling. Further pharmacological interventions reinforced a dual downstream signaling mechanism that involves the inhibition of the AC pathway and the activation of the PLC pathway, both of which converge on ERK1/2. Overall, these results suggest that the lactate/GPR81 pathway is essential for the critical aspects of HIIT-induced synaptic remodeling in the hippocampus and the enhancement of memory, supporting a GPR81-dependent dual-branch model that converges on ERK1/2 in a simplified *in vitro* context.

## Graphical Abstract

**Figure:**
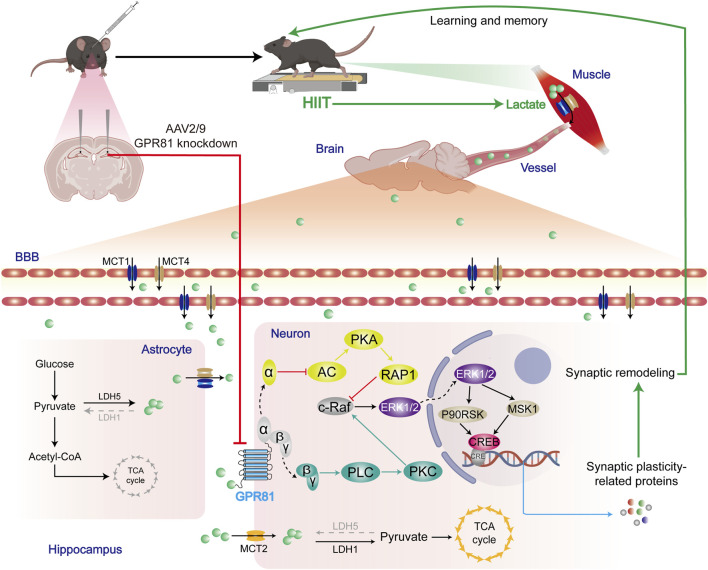
HIIT elevates blood and cerebral lactate levels *via* anaerobic muscle metabolism. Following the increase in lactate by HIIT, the lactate receptor GPR81 contributes to ERK1/2 activation *via* AC-RAP1 and PLC-PKC pathways. This pathway promotes the expression of selected synaptic plasticity-related proteins (e.g., SYN and BDNF) and thereby supports improved learning and memory.

## 1 Introduction

Cognitive abilities such as learning and memory are closely associated with the connections formed between neurons (Banasr et al., 2021). The regulation of synaptic connectivity, known as synaptic plasticity, serves as a fundamental mechanism by which the nervous system adjusts to environmental stimuli, thereby playing a pivotal role in establishing and sustaining cognitive functions (Dachtler et al., 2011). Synaptic plasticity involves both structural and functional alterations at synapses (von Bernhardi et al., 2017; Choi and Kaang, 2022). This process is modulated by various proteins related to synaptic plasticity that play roles in the formation, restructuring, and signaling of synapses (VanGuilder et al., 2011; Sun et al., 2021). The regulation of synaptic plasticity is linked to the activation of extracellular signal-regulated kinase 1/2 (ERK1/2)(Sweatt, 2001; Miningou and Blackwell, 2020) and the ensuing signaling pathways (Guo et al., 2020). When ERK1/2 is activated, it can lead to either an increase or decrease in the synthesis and expression of certain proteins, including those that are directly or indirectly involved in synapse formation and restructuring (Zhou et al., 2009). Additionally, ERK1/2 influences the activity of specific synaptic proteins through post-translational modifications, especially phosphorylation (Giachello et al., 2010; Maharana et al., 2013). These modifications allow neurons to adjust the strength of their connections based on experiences and environmental factors, which is crucial for the processes of learning and memory.

The hippocampus is a vital area of the brain essential for memory and learning processes. Enhancing synaptic plasticity in this region can significantly influence cognitive function development and preservation (Kim and Diamond, 2002; Neves et al., 2008). Recent findings in sports science highlight the remarkable benefits of consistent physical activity for cognitive wellbeing (Erickson et al., 2019), particularly focusing on high-intensity interval training (HIIT) due to its efficiency and cognitive advantages (Calverley et al., 2020). HIIT involves alternating short bursts of intense exercise with periods of lower intensity, and it has been shown to greatly improve synaptic plasticity in the hippocampus (Dos Santos et al., 2020). When compared to traditional steady-state aerobic workouts, HIIT offers superior enhancements to brain function (Constans et al., 2021). This superiority may stem from its more influential role in increasing cardiovascular function (Ramos et al., 2015), improving neuronal nutritional status, and enhancing the activity of neural networks associated with learning and memory (Afzalpour et al., 2015). Furthermore, HIIT may enhance cognitive abilities by triggering the release of neurotrophic factors and facilitating the creation of new neurons (Okamoto et al., 2021). Therefore, HIIT stands out as an effective form of exercise that supports brain health and cognitive function.

In studies examining the impact of HIIT on synaptic plasticity within the hippocampus, lactate has emerged as a key metabolic byproduct attracting significant research interest (Jacob et al., 2023; Lei et al., 2024). While traditionally considered a waste product of anaerobic metabolism in exercise physiology, recent studies have unveiled a broader biological function for lactate. It serves as an energy substrate for muscles and other tissues and plays a vital role in the functional regulation of the nervous system (Brooks, 2018; Brooks et al., 2022). Lactate has been identified as a potential signaling molecule in the brain, capable of influencing neuronal activity and synaptic plasticity (Magistretti and Allaman, 2018). Research indicates that lactate can boost neuronal excitability and enhance synaptic transmission efficiency (Herrera-Lopez et al., 2020). Additionally, it may support neuronal survival and the formation of synapses by regulating neuroprotective factors like brain-derived neurotrophic factor (BDNF) (El Hayek et al., 2019; Muller et al., 2020). Beyond its role in enhancing physical performance, lactate may also benefit cognitive abilities by fostering synaptic plasticity and optimizing neuronal operations. These positive effects are likely linked to its interaction with the G protein-coupled receptor 81 (GPR81), also referred to as hydroxycarboxylic acid receptor 1 (HCAR1), which is a Gi-type GPCR (G protein-coupled receptor) (Ge et al., 2008). Peripherally, GPR81 is highly expressed in adipose tissue, kidneys, skeletal muscles, heart, and other organs and tissues, reflecting its extensive role in systemic metabolic processes (Wu et al., 2006). In the central nervous system (CNS), the expression of GPR81 is predominantly localized in the neurons of the cerebral cortex and hippocampus, indicating its potential role in modulating signal transduction and neuronal activity within the CNS (Lauritzen et al., 2014; Morland et al., 2015).

Elevated lactate levels, often seen during vigorous exercise, can influence various physiological functions through the activation of GPR81. Research has shown that mice deficient in GPR81 exhibit delayed formation of cerebral microvessels (Chaudhari et al., 2022). These GPR81-lacking mice also demonstrate reduced vascular density compared to their counterparts with the intact GPR81 gene following hypoxic-ischemic injury (Chaudhari et al., 2022). The activation of GPR81 can be influenced by HIIT, which affects angiogenesis (Morland et al., 2017) and neurogenesis (Lambertus et al., 2021). Nonetheless, it remains unclear if the lactate/GPR81 pathway is responsible for the enhancement of hippocampal synaptic plasticity associated with HIIT. Peripheral studies suggest that GPR81 can produce unique physiological responses *via* the Gαi subunit (Sun et al., 2016) and the Gβγ complex (Wu et al., 2018). Moreover, it is established that Gi-type GPCRs can activate ERK1/2 through both pathways (May and Hill, 2008). Therefore, our research aimed to explore whether GPR81 activates ERK1/2 through both subunits in the hippocampus, facilitating synaptic plasticity. We examined the role of lactate/GPR81 in enhancing synaptic remodeling in the hippocampus as a result of HIIT. Additionally, we conducted *in vitro* studies to confirm the mediation process, concentrating on the roles of GPR81-coupled Gα and Gβγ subunits, their downstream signaling pathways, and the activation of ERK1/2.

## 2 Materials and methods

### 2.1 Animals

The 7-week-old male C57BL/6J mice were acquired from the Nanjing Model Animal Research Center and kept in the SPF animal laboratory (temperature of 22 °C ± 2 °C under a 12-h light/dark cycle) with unrestricted access to water and food.

The mice were divided into two batches according to the intervention schedule. Before the formal HIIT experiment, the first batch was used for a single-bout lactate verification test, including the sedentary control group (Con), the single aerobic exercise group (AE), and the single HIIT group (HIIT). he second batch comprised four groups: SC (scramble control, sedentary), SCH (scramble control + HIIT), GKD (GPR81 knockdown, sedentary), and GKDH (GPR81 knockdown + HIIT). This batch was used for the formal experiment, which consisted of a 6-week HIIT intervention.

### 2.2 Lentivirus and adeno-associated virus (AAV) vectors were constructed and packaged for GPR81 interference

The interference vectors used were pHBLV-U6-MCS-CMV-ZsGreen-PGK-PURO for lentivirus and pHBAAV2/9-U6-MCS-CMV-EGFP for AAV2/9. AAV2/9 was chosen due to its high efficiency in transducing hippocampal neurons following stereotaxic injection. In these vectors, shRNA expression was regulated by the U6 promoter, while the CMV promoter facilitated the expression of ZsGreen/EGFP for monitoring infection. Since the injection was directly into the hippocampal area, there was no need for additional cell type-specific promoters to achieve spatial specificity. The sequences for shRNA and the control scrambled variants can be found in Table 1. The construction, packaging, and purification of both lentivirus and AAV2/9 were carried out by Shanghai Hanheng Biotech.

**TABLE 1:** Sequences of lentiviral and AAV2/9 shRNA construct.

Groups	Sequences	
	Top strand	Bottom strand
NC	GAT​CCG​TTC​TCC​GAA​CGT​GTC​ACG​TAA​TTC​AAG​AGA​TTA​CGT​GAC​ACG​TTC​GGA​GAA​TTT​TTT​C	AAT​TGA​AAA​AAT​TCT​CCG​AAC​GTG​TCA​CGT​AAT​CTC​TTG​AAT​TAC​GTG​ACA​CGT​TCG​GAG​AAC​G
shRNA2	GAT​CCG​AAG​ATG​ACC​AAA​GTC​CAG​AGG​TTC​AAG​AGA​CCT​CTG​GAC​TTT​GGT​CAT​CTT​TTT​TTT​G	AAT​TCA​AAA​AAA​AGA​TGA​CCA​AAG​TCC​AGA​GGT​CTC​TTG​AAC​CTC​TGG​ACT​TTG​GTC​ATC​TTC​G
shRNA3	GAT​CCG​ACC​TGG​AAG​TCA​AGC​ACT​ATT​TTC​AAG​AGA​AAT​AGT​GCT​TGA​CTT​CCA​GGT​TTT​TTT​G	AAT​TCA​AAA​AAA​CCT​GGA​AGT​CAA​GCA​CTA​TTT​CTC​TTG​AAA​ATA​GTG​CTT​GAC​TTC​CAG​GTC​G
shRNA4	GAT​CCG​CCT​CTG​CAG​TAA​GAG​CTC​CAT​CGA​TTT​CAA​GAG​AAT​CGA​TGG​AGC​TCT​TAC​TGC​AGA​GGT​TTT​TTG	AAT​TCA​AAA​AAC​CTC​TGC​AGT​AAG​AGC​TCC​ATC​GAT​TCT​CTT​GAA​ATC​GAT​GGA​GCT​CTT​ACT​GCA​GAG​GCG
shRNA5	GAT​CCG​TCG​GTC​AAC​GTT​GTT​TGG​AGC​CTG​ATT​CAA​GAG​ATC​AGG​CTC​CAA​ACA​ACG​TTG​ACC​GAT​TTT​TTG	AAT​TCA​AAA​AAT​CGG​TCA​ACG​TTG​TTT​GGA​GCC​TGA​TCT​CTT​GAA​TCA​GGC​TCC​AAA​CAA​CGT​TGA​CCG​ACG

### 2.3 Hippocampal *in situ* stereotaxic surgery

To investigate the role of hippocampal GPR81 in mediating the effects of HIIT on synaptic plasticity, we conducted hippocampal orthotopic surgery. We injected packaged AAV2/9 (virus titer: 1.2 × 10^13^ vg/mL, injection volume: 1 μ/unilateral hippocampus) directly into the corresponding site (Anterior-Posterior: −2.3 mm, Medio-Lateral: ±1.9 mm, Dorsal-Ventral: −2.0 mm) to achieve hippocampal GPR81 knockdown (Supplementary Figures 1–4). The SC and SCH groups received injections of the scramble virus, while the GKD and GKDH groups received injections of the knockdown virus.

### 2.4 Exercise regimen and blood lactate measurement

Prior to the formal HIIT intervention, we performed a single-bout lactate verification test to confirm the lactate-elevating effect of HIIT. Animals completed either one session of aerobic exercise (AE) or one session of HIIT (using the same exercise protocol as in the subsequent formal intervention), and blood samples were collected from their tail tips immediately to measure the changes in blood lactate levels. Blood lactate levels are measured using the Lactate-Scout (EKF Co., Leipzig, Germany).

The AE regimen consists of a 1-h session with an intensity of 60% of the maximum exercise speed. The HIIT regimen is based on the research conducted by Morland et al. (2017). The program consists of three stages. The first stage is a 10-min warm-up training session at a speed of 10 m/min. Following the warm-up, there are 10 cycles, each consisting of 4 min of training at 85%–90% of the maximum exercise speed and 2 min at 40%–50%. Finally, there is a 5-min relaxation training session. Throughout the entire training process, the treadmill angle is set at 10° (Table 2). A test is conducted every other week to determine the maximum exercise capacity of the mice. The mice undergo a warm-up session lasting 10 min at 50% of their maximum speed. The speed is then increased by 2 m/min every 2 min. Exhaustion is determined when the mice receive five mild electric shocks within 15 s. The speed at which exhaustion occurs is recorded as the maximum movement speed of the mice for the next 2 weeks.

**TABLE 2:** HIIT regimen.

Week	Warm-up speed (m/min)	Warm-up duration (min)	Training speed (m/min)	Training duration (min)	Intermittent period speed (m/min)	Intermittent period duration (min)	Recovery speed (m/min)	Recovery duration (min)	Number of cycles	Session duration (min)
1–2	10	10	22–23	4	10	2	10	5	10	75
3–4			26.5–28.5		12–14.5					
5–6			30–33		12–15					

### 2.5 Y maze

During the spontaneous alternation assessment, a mouse was positioned at the end of one arm (50 cm × 18 cm × 35 cm) of the Y-shaped maze, oriented towards the base, and given the opportunity to explore for a duration of 8 min.

The memory recognition assessment consisted of two segments. Initially, a barrier was employed to block one section of the maze, allowing the mouse to roam freely for 10 min from the end of one of the open arms. After a 1-h break, the second segment took place. During this phase, the barrier was taken away, and the mouse was returned to the same arm it had explored previously, where it was allowed to investigate for 5 min.

### 2.6 Neuro-2a (N2a) cell culture

N2a cells were sourced from the Cell Bank of the Chinese Academy of Sciences. The cells were cultured in a constant temperature incubator at 37 °C with 5% CO_2_. The complete medium consisted of MEM (11090-081, Gibco, USA), 10% FBS (10099141C, Gibco), 1% GlutaMax (35050061, Gibco), 1% sodium pyruvate (11360070, Gibco), 1% NEAA (11140050, Gibco), and 1% penicillin-streptomycin solution (C0222, Beyotime, Shanghai, China).

### 2.7 Cellular intervention

Cellular intervention was conducted to investigate the effects of various interventions on GPR81 knockdown.

The lentivirus titer for GPR81 knockdown was 2 × 10^8^ TU/mL. The first verification involved a gradient of multiplicity of infection (MOI) set at 0, 5, 10, 18, and 24. The MOI gradient was set at 0, 10, 20, and 30 for the secondary verification.

We first established the time–concentration parameters for each pharmacological manipulation using four reagents: 3-chloro-5-hydroxybenzoic acid (CHBA; S5400, Selleck, USA), a GPR81 agonist; SCH772984 (S7101, Selleck, USA), an ERK1/2 inhibitor; forskolin (S2449, Selleck, USA), an adenyl cyclase (AC) agonist; and U73122 (S8011, Selleck, USA), a phospholipase C (PLC) inhibitor. For each reagent, pilot testing was performed in triplicate across three concentrations, yielding nine experimental conditions per intervention. Based on these optimization experiments, the final treatment conditions were set as follows: CHBA (Ga), 1 mM for 6 h; SCH772984 (Ei), 1 μM for 1 h; forskolin (Aa), 20 μM for 6 h; and U73122 (Pi), 5 μM for 6 h. Vehicle controls received an equivalent volume of DMSO.

### 2.8 Immunohistochemistry

Mice were anesthetized using isoflurane and underwent cardiac perfusion. Subsequently, their brains were soaked in 4% paraformaldehyde overnight. The tissues were then changed to a 30% sucrose solution and dehydrated for 7 days. After removing excess water, the tissues were embedded in OCT and sectioned using a freezing microtome (Leica, German) with a thickness of 40 μm. Next, they were treated with 0.5% Tritox for 15 min and soaked in PBS for 5 min, repeated thrice. To block non-specific binding, the tissues were incubated with 10% goat serum at 37 °C for 30 min. The GPR81 antibody (PA5-114741, 1:100, Thermo, USA) was incubated overnight at 4 °C. After soaking in PBS for 5 min, repeated three times, the tissues were incubated with a secondary antibody at a dilution of 1:300 for 1 h in the dark. Following another round of soaking in PBS for 5 min, repeated three times, the slides were mounted using a DAPI-containing mounting medium.

### 2.9 Golgi staining

To prepare the solution, 1 L of double distilled water was mixed with 10 g of gelatin and 0.5 g of potassium chromium sulfate. The mixture was heated until the ingredients dissolved, filtered, and cooled to room temperature. A clean glass slide was immersed in the solution and baked at 37 °C. The slide was then stored at 4 °C for future use. The FD Rapid Golgi StainTM Kit (PK401, FD NeuroTechnologies, USA) was utilized for sample preparation and staining, and the frozen section thickness was maintained at 100 μm.

### 2.10 Transmission electron microscope (TEM) observation

The hippocampal CA1 subregion was dissected into small blocks (∼1 mm^3^) and immediately fixed in electron microscopy fixative at 4 °C for 2–4 h. Samples were washed three times in 0.1 M phosphate buffer (PB, pH 7.4) and post-fixed in 1% osmium tetroxide in 0.1 M PB at room temperature for 2 h. Tissues were dehydrated through graded ethanol (50%, 70%, 80%, 90%, 95%, 100%, 100%) and acetone (100%, 100%), 15 min per step, infiltrated with acetone/812 resin (1:1) for 2-4 h, acetone/812 resin (1:2) overnight, and pure 812 resin for 5–8 h, embedded in fresh resin, and polymerized at 60 °C for 48 h. Ultrathin sections (60–80 nm) were cut using an ultramicrotome and stained with uranyl acetate and lead citrate (15 min each). Images were acquired using a transmission electron microscope for ultrastructural analysis, and synapse-related parameters in CA1—including postsynaptic membrane chord/arc length, synaptic cleft width, postsynaptic density (PSD) thickness/length, synapse number/density, and presynaptic synaptic vesicle number/density—were quantified.

### 2.11 Reverse transcription-quantitative PCR

Total RNA was extracted from tissues using TRIzol reagent (9109, Takara, Shiga, Japan). Reverse transcription was performed using the PrimeScript™ RT kit (RR047A, Takara). Real-time PCR detection was performed using the TB Green® Premix Ex Taq™ II (RR820A, Takara). The primer sequences were as follows: Hcar1 (GPR81): CTT​CCT​TTG​CCA​GAG​GTG​TTG​A (F); GGG​TCT​CAG​GGA​TAC​TCA​GGT​T (R). Gapdh (GAPDH): AGG​TCG​GTG​TGA​ACG​GAT​TTG (F); TGT​AGA​CCA​TGT​AGT​TGA​GGT​CA (R). Mapk1 (ERK): GGT​TGT​TCC​CAA​ATG​CTG​ACT (F); CAA​CTT​CAA​TCC​TCT​TGT​GAG​GG (R). Relative gene expression was quantified and normalized to Gapdh.

### 2.12 Western blotting

In the *in vivo* Western blotting experiments, the entire hippocampal tissue was used. To lyse hippocampal tissue and N2a cells, RIPA lysis buffer was used on ice. 200 μL was added to a single sample for hippocampal tissue, while 100 μ was added for N2a cells. After sonication, the mixture was centrifuged at 4 °C, 14,000 rpm/min for 15 min. The supernatant was collected, and the protein concentration was determined using the BCA protein assay kit to prepare WB samples. A 10% PAGE gel (PG112/PG113, Epizyme, Shanghai, China) was used for gel electrophoresis, with a minimum of 20 µg of protein loaded per well. The electrophoresis transfer conditions were set at 70–110 V, 300 mA/70 min (Bio-Rad, USA). After a 15-min blocking step (PS108P, Epizyme, Shanghai, China), the samples were incubated with primary antibodies overnight at 4 °C. GPR81, PA5-114741, Thermo; AC (55067-1-AP, Proteintech), PKA (4782S, CST), RAP1 (2399S, CST), PKC (SC-80 Santa), PLC (14247S, CST), Raf1 (9422S, CST), MSK1 (3489S, CST), P90RSK (9355S, CST), ERK (4370T, CST), CREB (9197S, CST), c-Fos (2250S, CST), Zif268 (4154S, CST), NMDAR (5704S, CST), SYN (4329S, CST), BDNF (28205-1-AP, Proteintech), ARC (16290-1-AP, Proteintech), PSD95 (3450S, CST) p-Raf1 (9421S, CST), p-ERK (4370T, CST), p-MSK (9595S, CST), p-P90RSK (11989S, CST), p-CREB (9198S, CST), Tubulin (10068-1-AP, Proteintech), HRP-conjugated Affinipure Goat Anti-Rabbit IgG (H + L) (SA00001-2, Proteintech), HRP-conjugated Affinipure Goat Anti-Mouse IgG (H + L) (SA00001-1, Proteintech). Relative protein expression was quantified by densitometry and normalized to Tubulin. Phosphorylated proteins were further normalized to their corresponding total protein levels.

### 2.13 Statistical analysis

Data are presented as mean ± standard deviation (SD). Statistical analyses were performed using SPSS 22.0. For comparisons between two groups, an independent-samples t-test was used. For experiments with predefined experimental groups (≥3 groups), one-way ANOVA followed by appropriate *post hoc* multiple-comparison tests was applied to address specific group-based biological questions. For Sholl analysis, a two-way repeated measures ANOVA was conducted with distance as the within-subject factor and group as the between-subject factor; Greenhouse–Geisser correction was used when sphericity was violated. A two-tailed *p* value <0.05 was considered statistically significant.

## 3 Results

### 3.1 Construction of GPR81 knockdown mouse model and the promotive effect of HIIT on GPR81

**FIGURE 1:**
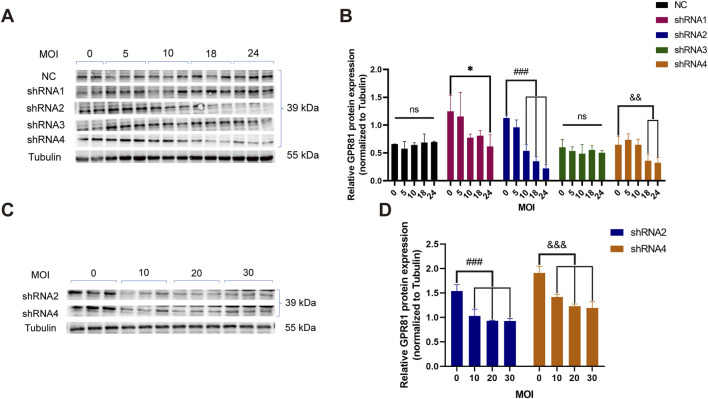
Screening of GPR81 knockdown sequences in N2a cells. **(A,B)** Representative Western blotting images and quantitative analysis demonstrating the interference in GPR81 expression in N2a cells by lentiviruses containing the four target shRNA sequences at different MOI gradients. **(C,D)** Representative Western blotting images and quantitative analysis of GPR81 protein expression following secondary validation of shRNA2 and shRNA4 in viruses at four MOI gradients. The Tubulin bands shown are representative. All blot images are provided in Supplementary Figure 6. Protein expression levels were normalized to Tubulin. NC, negative control. MOI, multiplicity of infection. For comparisons among multiple groups, one-way ANOVA was applied. The data are presented as mean ± SD. *p < 0.05, **p < 0.01, ***p < 0.001 vs*.* MOI = 0 within the shRNA1 group. ^###^ *p* < 0.001 vs*.* MOI = 0 within the shRNA2 group. ^&^ *p* < 0.05, ^&&^ *p* < 0.01, ^&&&^ *p* < 0.001 vs*.* MOI = 0 within the shRNA4 group. ns, no significant difference. For comparisons among multiple groups, one-way ANOVA was applied.

To identify an efficient shRNA targeting GPR81, N2a cells were infected with lentiviruses expressing four candidate shRNAs or a negative control (NC) at MOIs of 0, 5, 10, 18, and 24, followed by Western blotting analysis. GPR81 protein levels in the NC group were not significantly altered across MOIs, indicating that viral dose alone did not affect baseline expression (Figures 1A,B). In contrast, shRNA2 and shRNA4 produced a clear MOI-dependent reduction in GPR81 protein, whereas shRNA1 and shRNA3 showed no significant effects (Figures 1A,B). Notably, shRNA2 achieved significant knockdown at MOI 10 (0.537 ± 0.112, *p* < 0.001), 18 (0.350 ± 0.09, *p* < 0.001) and 24 (0.224 ± 0.068, *p* < 0.001) and displayed the strongest overall suppression, while shRNA4 also reduced GPR81 at higher MOIs but with comparatively lower efficiency (Figure 1B). Occasional minor band imperfections (e.g., localized bubble artifacts) were observed during Western blotting imaging (Figure 1A). Excluding the affected region did not alter the quantified results or statistical conclusions, which were reproducible across independent replicates (Supplementary Figure 6).

These results were confirmed in an independent experiment using shRNA2 and shRNA4 at MOIs of 0, 10, 20, and 30, which consistently showed significant, dose-dependent decreases in GPR81 protein at MOI ≥10 (*p* < 0.001) (Figures 1C,D). Based on knockdown efficacy and reproducibility, shRNA2 was selected for subsequent AAV2/9 vector construction and packaging for GPR81 knockdown.

**FIGURE 2:**
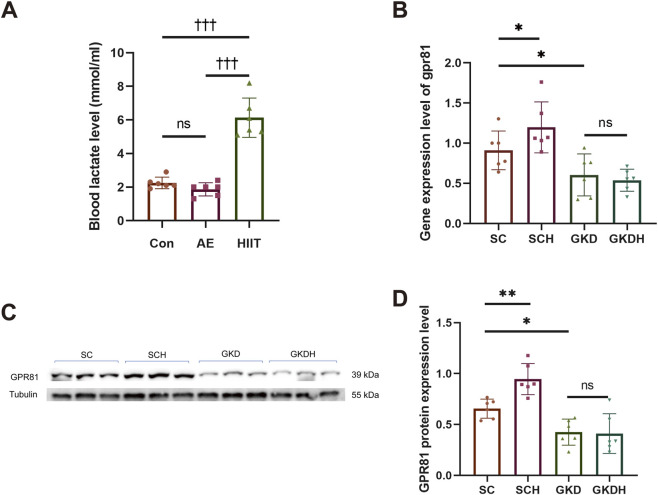
Successful construction of a hippocampal GPR81 knockdown model. **(A)** Changes in blood lactate levels in mice after a single AE and HIIT session. **(B)** Quantitative analysis of gpr81 gene expression in the hippocampus. **(C,D)** Representative Western blotting images and quantitative analysis of GPR81 protein expression in the hippocampus. Protein expression levels were normalized to Tubulin. Relative gene expression was quantified and normalized to Gapdh. For comparisons among multiple groups, one-way ANOVA was applied. The data are presented as mean ± SD. *n* = 6. ^†††^ *p* < 0.001 vs*.* HIIT group. *p < 0.05, **p < 0.01 vs*.* SC group. ns, no significant difference; SC, scramble control, sedentary; SCH, scramble control + HIIT; GKD, GPR81 knockdown, sedentary; GKDH, GPR81 knockdown + HIIT.

Prior to the formal HIIT intervention, blood lactate was measured after a single bout of aerobic exercise (AE) and a single bout of HIIT to verify the lactate-elevating effect of HIIT. The results showed no significant difference between the Con and AE groups. In contrast, the HIIT group (6.133 ± 1.174 mM) exhibited a markedly higher blood lactate level than both the Con group (2.25 ± 0.345 mM) (*p* < 0.001) and the AE group (1.867 ± 0.398 mM) (*p* < 0.001), demonstrating that a single HIIT session robustly induces lactate accumulation and confirming its effectiveness in elevating lactate for the subsequent experiments (Figure 2A).

**FIGURE 3:**
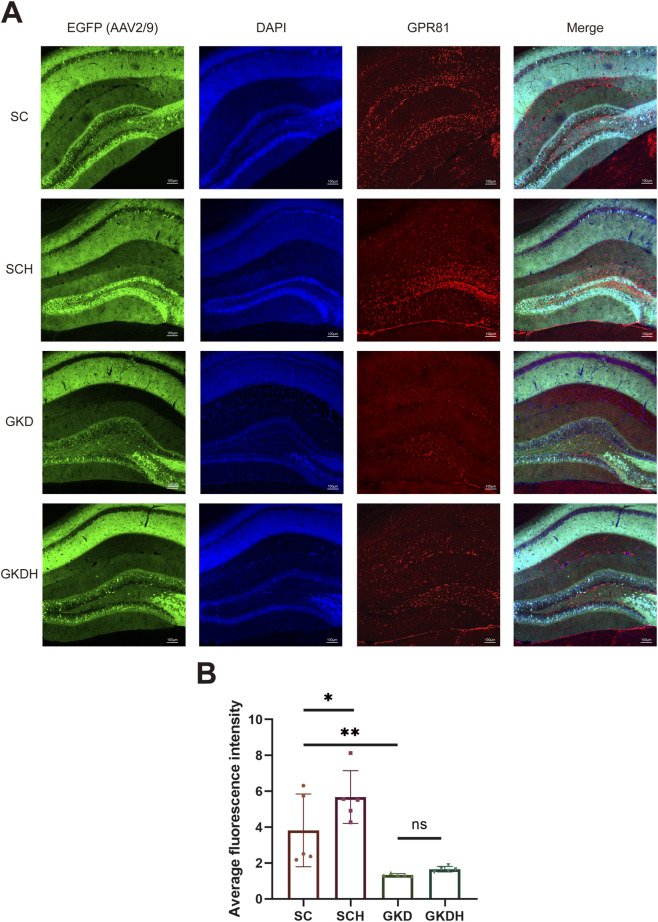
Immunofluorescence staining of hippocampal GPR81 in different groups. **(A)** Horizontally, images show hippocampal region staining for AAV2/9 infection (EGFP, green), DAPI staining (blue), GPR81 (red), and composite images (Merge). Vertically, the groups are SC, SCH, GKD, and GKDH, respectively. The scale bar is 100 μm. **(B)** Quantitative analysis of the average fluorescence intensity among the groups. Fluorescence intensity was quantified and expressed as arbitrary units (a.u.). For comparisons among multiple groups, one-way ANOVA was applied. The data are presented as mean ± SD. *n* = 5. *p < 0.05, **p < 0.01 vs*.* the SC group. ns, no significant difference; SC, scramble control, sedentary; SCH, scramble control + HIIT; GKD, GPR81 knockdown, sedentary; GKDH, GPR81 knockdown + HIIT.

The results showed that stereotaxic injection of AAV2/9 into the hippocampus significantly reduced hippocampal GPR81 gene expression and protein levels. Moreover, HIIT led to a significant increase in hippocampal GPR81 gene (Figure 2B) and protein expression levels (Figures 2C,D). However, the exercise-induced increase in hippocampal GPR81 levels was hindered after GPR81 knockdown (Figures 2B–D). Immunofluorescence staining further corroborated these molecular findings: compared with SC, SCH displayed stronger GPR81 immunoreactivity (red) in the hippocampal region examined, whereas both GKD and GKDH showed markedly weaker GPR81 signals despite comparable EGFP expression (Figure 3A). Quantification of average fluorescence intensity (arbitrary units, a.u.) confirmed a significant increase in SCH (5.68 ± 1.468) *versus* SC (3.822 ± 2.025) (*p* < 0.05), significant reductions in GKD (1.336 ± 0.073) *versus* SC (*p* < 0.01), and no significant difference between GKD and GKDH (1.658 ± 0.151) (Figure 3B).

### 3.2 GPR81-mediated enhancement of synaptic structures in hippocampal neurons of mice by HIIT

**FIGURE 4:**
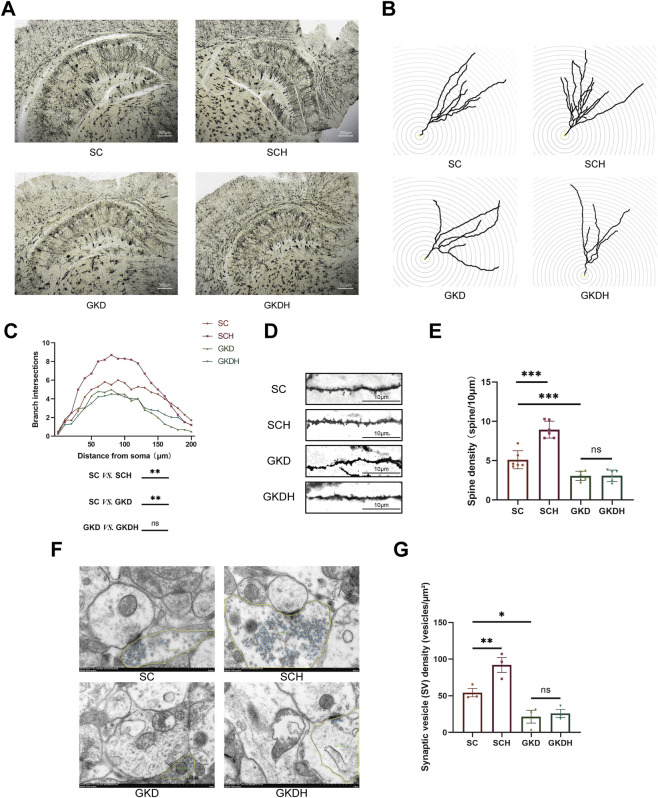
GPR81-mediated enhancement of synaptic structures in hippocampal neurons of mice by HIIT. **(A)** Representative Golgi staining images of hippocampal neurons from each group. The images were magnified at ×4; the scale bar represents 200 μm. **(B,C)** Representative images and quantitative analysis of dendritic branching in hippocampal DG neurons of each group using Sholl analysis. **(D,E)** Representative images and quantitative analysis of dendritic spine density in hippocampal DG neurons of each group. The images were magnified at ×630, and the scale bar represents 10 μm. **(F,G)** Representative TEM images of presynaptic terminals from each group, with synaptic vesicles highlighted (blue) and the presynaptic terminal area outlined (yellow), and the corresponding quantitative analysis of synaptic vesicle (SV) density (vesicles/µm^2^).The scale bar represents 500 nm. For comparisons among multiple groups, one-way ANOVA was applied. For Sholl analysis, a two-way repeated measures ANOVA was conducted with distance as the within-subject factor and group as the between-subject factor; Greenhouse-Geisser correction was used when sphericity was violated. The data are presented as mean ± SD. *n* = 6 (Golgi), *n* = 3 (TEM). *p < 0.05, **p < 0.01, ***p < 0.001 vs*.* the SC group. ns, no significant difference; SC, scramble control, sedentary; SCH, scramble control + HIIT; GKD, GPR81 knockdown, sedentary; GKDH, GPR81 knockdown + HIIT.

Dendritic complexity was assessed by Sholl analysis using a two-way repeated-measures ANOVA (within-subject factor: distance; between-subject factor: group). Mauchly’s test indicated violation of sphericity (*W* = 0.000, *p* < 0.001); therefore, Greenhouse–Geisser–corrected results were reported. A significant main effect of distance was observed (*F* (3.161, 63.217) = 47.057, *p* < 0.001), together with a significant distance × group interaction (*F* (9.483, 63.217) = 2.779, *p* < 0.01), indicating group-dependent differences in Sholl intersections across radii. The between-subject effect of group was also significant (*F* (3, 20) = 26.669, *p* < 0.001, ηp^2^ = 0.800). Post hoc comparisons based on estimated marginal means (±SE, averaged across radii) showed increased dendritic complexity (number of intersections) after HIIT (SCH: 5.458 ± 0.237 vs. SC: 4.133 ± 0.237, *p* < 0.01), whereas GPR81 knockdown reduced dendritic complexity (GKD: 2.875 ± 0.237 vs. SC, *p* < 0.01). Notably, HIIT did not rescue the reduction under GPR81 knockdown (GKDH: 2.917 ± 0.237) (Figures 4A–E).

TEM analyses showed that synapses in the SC group displayed well-preserved ultrastructure, including clearly delineated presynaptic and postsynaptic membranes, an identifiable synaptic cleft, and a prominent PSD (Figure 4F). In the SCH group, presynaptic boutons exhibited a visibly denser accumulation of synaptic vesicles (SVs) compared with SC (Figure 4F). Quantitatively, SV density (vesicles/µm^2^) was significantly increased in SCH (92.13 ± 17.38) relative to SC (54.35 ± 9.76) (*p* < 0.01) (Figure 4G), indicating that HIIT was associated with enhanced presynaptic vesicle availability at hippocampal synapses. In contrast, GPR81 knockdown groups (GKD and GKDH) showed markedly lower SV abundance in representative boutons (Figure 4F). Morphometric analysis confirmed that SV density was significantly reduced in GKD (21.55 ± 15.19) compared with SC (*p* < 0.05) (Figure 4G). Importantly, HIIT failed to restore SV density under GPR81 knockdown, as no significant difference was detected between GKD and GKDH (Figure 4G). To further evaluate postsynaptic morphology, PSD thickness and PSD length were quantified (Supplementary Figure 5). No significant group differences were observed for either PSD thickness or PSD length (both ns), suggesting that the primary ultrastructural change detected here was presynaptic (SV density) rather than postsynaptic PSD morphology.

### 3.3 GPR81-mediated enhancement of learning and memory in mice by HIIT

**FIGURE 5:**
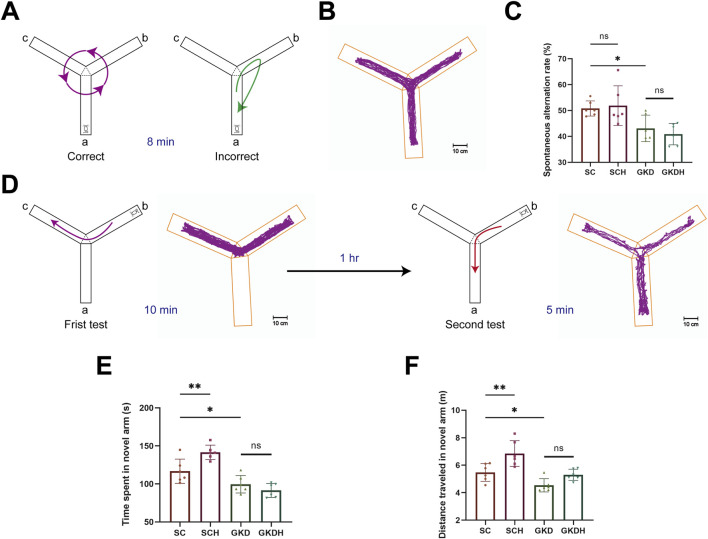
GPR81 mediates the enhancements of spatial working and recognition memory in mice by HIIT. **(A)** Spontaneous alternation test. A successful alternation is counted when a mouse enters different arms consecutively, with other scenarios considered errors. **(B)** Heatmap of mouse activity in the spontaneous alternation test. **(C)** Quantitative analysis of the SAT in each group. **(D)** Memory recognition test. In the first test, arm a is blocked, allowing the mouse to explore arms b and c for 10 min freely; 1 h later, arm a is opened for the second test. **(E,F)** Quantitative analysis of the time and distance explored in the novel arm by each group of mice in the memory recognition test. For comparisons among multiple groups, one-way ANOVA was applied. The data are presented as mean ± SD. *n* = 6. *p < 0.05, **p < 0.01 vs*.* the SC group. ns, no significant difference; SC, scramble control, sedentary; SCH, scramble control + HIIT; GKD, GPR81 knockdown, sedentary; GKDH, GPR81 knockdown + HIIT.

Compared with the SC group, GPR81 knockdown impaired Y-maze performance. Spontaneous alternation rate did not differ between SC (50.79% ± 2.94%) and SCH (51.87% ± 7.69%), indicating that HIIT alone did not affect baseline alternation behavior. In contrast, the alternation rate was significantly reduced in the GKD group (43.08% ± 5.09%) compared with SC (*p* < 0.05). Importantly, HIIT failed to improve spontaneous alternation under GPR81 knockdown, as no significant difference was observed between GKD and GKDH (40.87% ± 4.13%) (Figures 5A–C).

In the memory recognition test, mice in the SCH group exhibited a marked increase in both exploration time and distance in the novel arm (time: 141.63 ± 9.42 s; distance: 6.85 ± 0.94 m) compared with the SC group (time: 116.78 ± 15.96 s; distance: 5.48 ± 0.64 m) (*p* < 0.01), indicating that HIIT significantly enhanced recognition memory (Figures 5C,E,F). In contrast, GPR81 knockdown markedly reduced novel arm exploration, as evidenced by significantly decreased exploration time and distance in the GKD group (time: 99.60 ± 11.52 s; distance: 4.54 ± 0.48 m) relative to the SC group (*p* < 0.05) (Figures 5E,F). Importantly, HIIT failed to rescue this deficit under GPR81 knockdown conditions, as no significant differences were observed between the GKD and GKDH groups (time: 91.43 ± 9.24 s; distance: 5.30 ± 0.41 m) (Figures 5C,E,F).

### 3.4 Effects of GPR81 knockdown and HIIT on AC-RAP1 and PLC-PKC pathways, ERK1/2 activation, and synaptic plasticity-related proteins in the hippocampus

**FIGURE 6:**
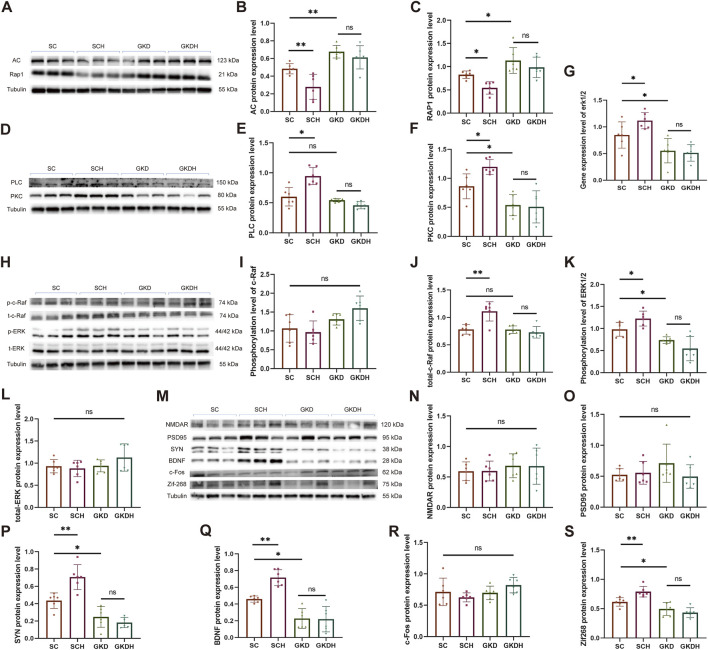
GPR81 mediates the regulation of HIIT on the AC-RAP1 and PLC-PKC pathways and ERK1/2 activation in the hippocampus of mice, as well as the expression of synaptic plasticity-related proteins. **(A,D,H,M)** Representative Western blotting images. **(B,C,E,F)** Quantitative analysis of AC, RAP1, PLC, and PKC protein expression levels in the hippocampus of different groups. **(I–L)** Quantitative analysis of protein expression levels of phosphorylated/total c-Raf and ERK1/2 in the hippocampus of different groups. **(G)** Quantitative analysis of gene expression levels of ERK1/2 in the hippocampus of each group. *p < 0.05, **p < 0.01 vs*.* the SC group. ns, no significant difference. **(N–S)** Quantitative analysis of protein expression levels of NMDAR, PSD95, SYN, BDNF, c-FOS, and Zif268 in the hippocampus of mice from different groups. Protein expression levels were normalized to Tubulin. Phosphorylated proteins were further normalized to their corresponding total protein levels. Relative gene expression was quantified and normalized to Gapdh. For comparisons among multiple groups, one-way ANOVA was applied. The data are presented as mean ± SD. *n* = 6. *p < 0.05, **p < 0.01 vs*.* the SC group. ns, no significant difference. SC, scramble control, sedentary; SCH, scramble control + HIIT; GKD, GPR81 knockdown, sedentary; GKDH, GPR81 knockdown + HIIT.

HIIT significantly reduced the protein expression of AC (SCH: 0.279 ± 0.141 vs. SC: 0.483 ± 0.058, *p* < 0.01) and Repressor/Activator Protein 1 (RAP1) (SCH: 0.543 ± 0.133 vs. SC: 0.825 ± 0.08, *p* < 0.05) in the hippocampus (Figures 6A–C) while simultaneously enhancing the expression of PLC (SCH: 0.946 ± 0.143 vs. SC: 0.600 ± 0.154, *p* < 0.05) and protein kinase C (PKC) (SCH: 1.197 ± 0.128 vs. SC: 0.862 ± 0.215, *p* < 0.05) (Figures 6D–F). These results indicate that HIIT can concurrently inhibit the Gα-AC-RAP1 signaling pathway and activate the Gβγ-PLC-PKC signaling pathway. Furthermore, when hippocampal GPR81 was knocked down, the protein expression of AC (GKD: 0.677 ± 0.0712 vs. SC, *p* < 0.01) and RAP1 (GKD: 1.132 ± 0.277 vs. SC, *p* < 0.05) increased, while the expression of PKC (GKD: 0.537 ± 0.184 vs. SC, *p* < 0.05) decreased. Notably, the effects of HIIT on these proteins were nullified after the knockdown of GPR81 (Figures 6A–F).

Both the AC-RAP1 and PLC-PKC signaling pathways can activate the MAPK signaling pathway through c-Raf, which ultimately affects the phosphorylation level of ERK1/2 (May and Hill, 2008). Our study found that while the phosphorylation level of c-Raf remained unchanged, the total protein level significantly increased after HIIT (SCH: 1.111 ± 0.176 vs. SC: 0.781 ± 0.087, *p* < 0.01). However, when GPR81 was knocked down, the protein expression level of total-c-Raf returned to normal (GKD: 0.778 ± 0.068 vs. SC, ns), and the impact of exercise was nullified (GKDH: 0.728 ± 0.106 vs. GKD, ns) (Figures 6H–J). In addition, we investigated the gene expression and phosphorylated protein expression levels of ERK1/2. The results showed that HIIT significantly increased the gene and phosphorylated protein expression levels of ERK1/2 compared to the SC group (*p* < 0.05). However, when GPR81 was knocked down, there was a significant decrease in these levels (*p* < 0.05), and the positive effects of exercise were lost. There were no significant differences in the total protein levels among the groups (Figures 6G,H,K,L).

Compared to the SC group, HIIT significantly enhanced the protein expression of synaptic plasticity-related proteins SYN (SCH: 0.707 ± 0.143 vs. SC: 0.435 ± 0.090, *p* < 0.01) and BDNF (SCH: 0.716 ± 0.095 vs. SC: 0.459 ± 0.037, *p* < 0.01) and the immediate early genes (IEGs) like Zif268 (SCH: 0.789 ± 0.09 vs. SC: 0.615 ± 0.073, *p* < 0.01). In mice, a marked decrease in SYN (GKD: 0.247 ± 0.120 vs. SC, *p* < 0.05), BDNF (GKD: 0.227 ± 0.118 vs. SC, *p* < 0.05), and Zif268 (GKD: 0.494 ± 0.11 vs. SC, *p* < 0.05) expression in the hippocampus was observed following GPR81 knockdown. However, HIIT did not elevate the expression levels of these proteins in the GKDH group compared to the GKD group (Figures 6M,P,Q,S). Furthermore, the protein expression levels of NMDAR, PSD95, and c-Fos were unaffected by GPR81 knockdown or HIIT (Figures 6M–O,R).

### 3.5 Time-concentration effects and expression of downstream pathway proteins by GPR81 agonist (Ga), AC agonist (Aa), PLC inhibitor (Pi), and ERK1/2 inhibitor (Ei)

**FIGURE 7:**
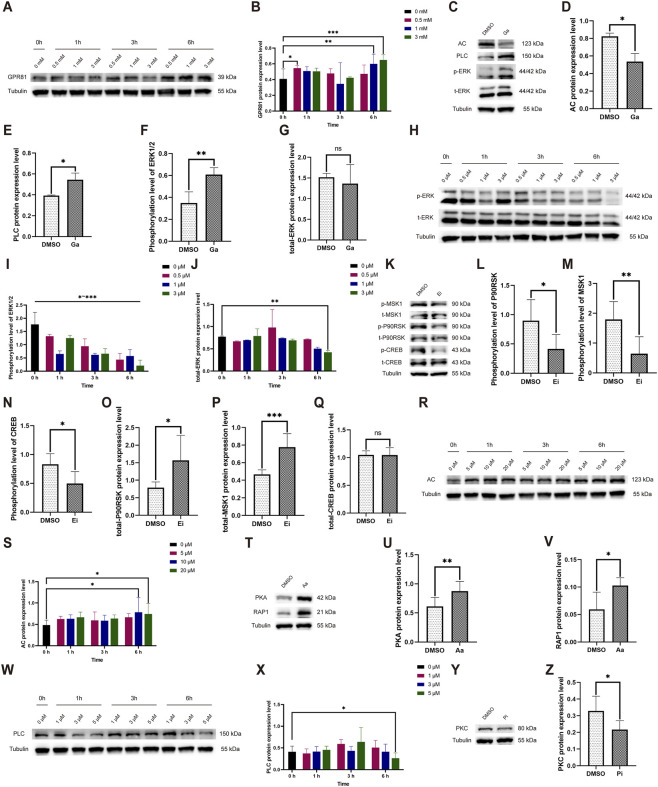
Exploration of the time-concentration effect of Ga, Aa, Pi, Ei and changes in pathway protein expression levels following formal intervention. **(A,H,R,W)** Representative Western blotting images showing GPR81, AC, and PLC protein expression, as well as phosphorylated/total ERK1/2 expression levels at various time and concentration levels. **(B,I,J,S,X)** Quantitative analysis of GPR81, AC, and PLC protein expressions, and phosphorylated/total ERK1/2 protein expression across different time and concentration levels. **(C,K,T,Y)** Representative Western blotting images of AC, PLC, PKA, RAP1, PKC, and phosphorylated/total ERK1/2, MSK1, P90RSK, and CREB protein expression levels. **(D–G,L–Q,U,V,Z)** Quantitative analysis of AC, PLC, PKA, RAP1, PKC, and phosphorylated/total ERK1/2, MSK1, P90RSK, and CREB protein expression levels. Protein expression levels were normalized to Tubulin. Phosphorylated proteins were further normalized to their corresponding total protein levels. For comparisons between two groups, an independent-samples t-test was used. For comparisons among multiple groups, one-way ANOVA was applied. The data are presented as mean ± SD. *n* = 3. *p < 0.05, **p < 0.01, ***p < 0.001 vs*.* the DMSO group. ns, no significant difference; Ga, GPR81 agonist; Ei, ERK1/2 inhibitor; Aa, AC agonist; Pi, PLC inhibitor.

Our results demonstrated that treating the cells with Ga at concentrations of 0.5 mM for 1 h (0.545 ± 0.034 vs. 0 mM/0 h: 0.408 ± 0.127), 1 mM for 6 h (0.599 ± 0.123 vs. 0 mM/0 h), and 3 mM for 6 h (0.649 ± 0.068 vs. 0 mM/0 h) significantly increased the levels of GPR81 protein expression (Figures 7A,B). After considering the concentration of the solvent DMSO, we determined that a duration of 6 h and a concentration of 1 mM would be suitable for the formal intervention. In the formal intervention, compared to the control group, the GPR81 agonist significantly reduced AC protein expression and upregulated PLC protein expression (Figures 7C–E). Simultaneously, while the total protein level of ERK1/2 remained constant, the phosphorylated protein expression level significantly increased (Figures 7C,F,G). Combined with the changes in AC and PLC after the knockdown of GPR81 *in vivo*, these findings suggest that activation or deficiency of GPR81 can regulate downstream AC-RAP1 and PLC-PKC in the CNS, thereby influencing ERK1/2 phosphorylation levels.

The Ei significantly reduced the phosphorylation level of ERK1/2 at all tested times and concentrations. The formal experiment selected an intervention time of 1 h with a concentration of 1 μM (0.656 ± 0.114 vs. 0 μM/0 h: 1.768 ± 0.455) (Figures 7H–J). After the formal intervention, the Ei group exhibited a significant decrease in phosphorylated levels of MSK1/P90RSK and CREB, along with a significant increase in the total protein level of MSK1/P90RSK, and no significant change in the total protein level of CREB (Figures 7K–Q).

The results indicated that the Aa was most effective at a concentration of 10 (0.783 ± 0.342 vs. 0 μM/0 h: 0.486 ± 0.113) and 20 μM (0.741 ± 0.245 vs. 0 μM/0 h) after 6 h (Figures 7R,S). For the formal experiment, we chose an intervention time of 6 h with a concentration of 20 μM. Upon AC activation during the formal intervention, the Aa group exhibited a significant increase in downstream PKA and RAP1 protein expression (Figures 7T–V).

The Pi demonstrated optimal efficacy at a concentration of 5 μM following a 6-h incubation period (0.262 ± 0.121 vs. 0 μM/0 h: 0.408 ± 0.131), which was the parameter chosen for the formal experimental setup (Figures 7W,X). Under the formal intervention, the Pi group demonstrated a significant decrease in PKC protein expression (Figures 7Y,Z).

### 3.6 GPR81 modulates ERK1/2 through the AC-RAP1 and PLC-PKC pathways and regulates synaptic plasticity-related proteins in N2a cells

**FIGURE 8:**
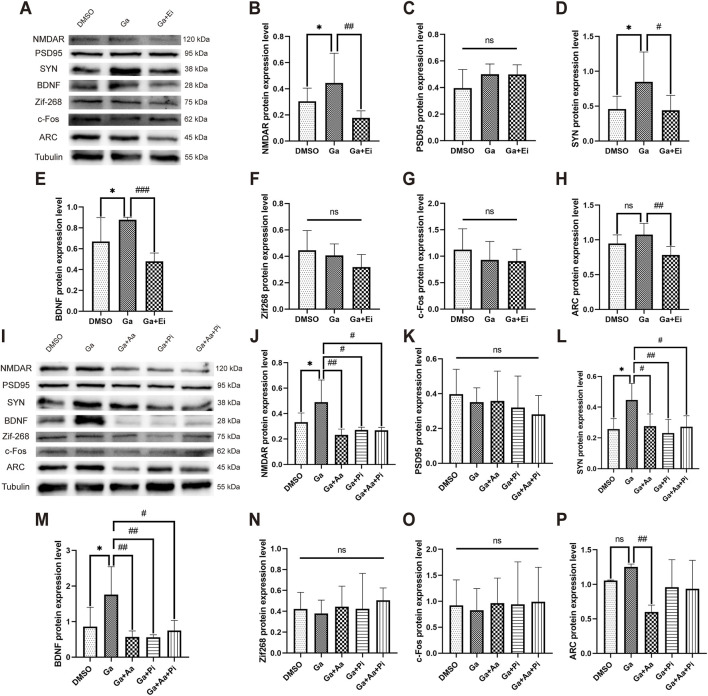
GPR81-induced modulation of ERK1/2 *via* AC-RAP1 and PLC-PKC pathways regulates synaptic plasticity-related protein expression in N2a cells. **(A,I)** Representative Western blotting images. **(B–H,J–P)** Quantitative analysis of NMDAR, PSD95, SYN, BDNF, Zif268, c-Fos, and ARC protein expression levels. Protein expression levels were normalized to Tubulin. Phosphorylated proteins were further normalized to their corresponding total protein levels. For comparisons among multiple groups, one-way ANOVA was applied. The data are presented as mean ± SD. *p < 0.05 vs*.* the DMSO group. *n* = 3. ^#^ *p* < 0.05, ^##^ *p* < 0.01, ^###^ *p* < 0.001 vs*.* the Ga group. ns, no significant difference. Ga, GPR81 agonist; Ei, ERK1/2 inhibitor; Aa, AC agonist; Pi, PLC inhibitor.

The results showed that GPR81 agonist (CHBA, Ga) significantly increased the expression of NMDAR (Ga: 0.444 ± 0.226 vs. DMSO: 0.304 ± 0.102, *p* < 0.05) (Figures 8A,B), SYN (Ga: 0.848 ± 0.384 vs. DMSO: 0.459 ± 0.184, *p* < 0.05) (Figures 8A,D), and BDNF (Ga: 0.878 ± 0.024 vs. DMSO: 0.667 ± 0.231, *p* < 0.05) (Figures 8A,E) but had no apparent effect on PSD95 and IEGs (Figures 8A,F–H). On the other hand, the combined use of Ga and ERK1/2 inhibitor (Ga + Ei group) significantly reduced the expression of NMDAR (Ga + Ei: 0.177 ± 0.054 vs. Ga, *p* < 0.01), SYN (Ga + Ei: 0.440 ± 0.214 vs. Ga, *p* < 0.05), BDNF (Ga + Ei: 0.479 ± 0.080 vs. Ga, *p* < 0.001), and the immediate early gene ARC (Ga + Ei: 0.784 ± 0.122 vs. Ga, *p* < 0.01) without causing significant effects on PSD95, Zif268, and c-Fos expression (Figures 8A–H).

To investigate whether GPR81 promotes the expression of synaptic plasticity–related proteins *via* the AC-RAP1 and PLC-PKC pathways, Ga, Aa (AC agonist), and Pi (PLC inhibitor) were applied either individually or in combination. The results for the Ga group were consistent with the previous section, showing that Ga significantly increased the expression of NMDAR, SYN, and BDNF compared to the DMSO group. However, it had no apparent effect on PSD95 and IEGs. Compared to the Ga group, the Ga + Aa, Ga + Pi, and Ga + Aa + Pi groups exhibited a significant reduction in the expression of NMDAR (*p* < 0.05–0.01), SYN (*p* < 0.05–0.01), BDNF (*p* < 0.05–0.01), and the immediate early gene ARC. However, no significant effects were observed on PSD95, Zif268, and c-Fos expression (Figures 8I–P).

## 4 Discussion

This research investigated the necessity of the lactate/GPR81 pathway for essential aspects of hippocampal synaptic remodeling and memory enhancement induced by HIIT, while also analyzing its downstream signaling characteristics. HIIT elevated GPR81 gene and protein expression and was accompanied by altered downstream AC and PLC expression *in vivo*. These changes were associated with increased ERK1/2 phosphorylation and higher levels of the synaptic remodeling-related proteins BDNF and SYN, as well as the activity-dependent gene Zif268, whereas NMDAR, PSD95 and c-Fos were not significantly changed. Consistent with these molecular observations, HIIT increased dendritic spine density and presynaptic synaptic vesicle (SV) density, without detectable changes in PSD thickness or length and improved learning and memory, and these structural and behavioral benefits were attenuated after GPR81 knockdown. In N2a cells, activation of GPR81 increased ERK1/2 phosphorylation and upregulated SYN and BDNF together with low-level NMDAR expression, while leaving Zif268 and c-Fos unchanged. In this simplified *in vitro* system, pharmacological inhibition of ERK1/2 and manipulation of AC and PLC provided support for a dual-branch downstream working model in which inhibition of the Gα-AC-RAP1 pathway and activation of the Gβγ-PLC-PKC pathway may converge on ERK1/2-P90RSK/MSK1-CREB signaling to regulate synaptic plasticity-related proteins.

The elevation in lactate levels directly correlates with the intensity of exercise. When oxygen consumption during exercise increases and gradually approaches the individual lactate threshold, there is a significant production of lactate, exceeding the clearance efficiency of the body. Consequently, HIIT markedly elevates lactate levels within the organism, in contrast to moderate-intensity aerobic exercise (Beneke et al., 2011; Hu et al., 2021). Lactate produced by skeletal muscles serves as a metabolic byproduct and an energy substrate in different tissues and organs like the heart, liver, and kidneys. This process is supported by the circulatory system and facilitated by monocarboxylate transporters (MCTs)(Brooks, 2020). In the brain, the role of lactate as a source of energy is notably more prominent. Under physiological conditions, lactate dehydrogenase (LDH) isozyme five within astrocytes converts pyruvate to lactate (Bittar et al., 1996). Following this, lactate enters neurons through the MCT2 and is converted back to pyruvate primarily by LDH1 (Pierre and Pellerin, 2005). It subsequently contributes to energy production by entering the tricarboxylic acid cycle. This process forms the fundamental framework of the astrocyte-neuron lactate shuttle (ANLS) (Bonvento and Bolanos, 2021). During exercise, the contribution of lactate to the energy supply within the brain further increases, reaching up to 27.8% at its peak (Smith and Ainslie, 2017). Blood lactate levels were assessed after a single bout of aerobic exercise or HIIT. Moderate-intensity aerobic exercise did not increase lactate above resting levels, whereas HIIT markedly elevated blood lactate. Our previous study directly quantified hippocampal lactate after a 6-week HIIT protocol and showed a significant increase from 1.39 ± 0.25 mM in controls to 1.90 ± 0.52 mM in the HIIT group (Hu et al., 2021). This finding suggests an increased contribution of lactate to brain energy metabolism under HIIT conditions.

In addition to providing energy, lactate serves as a signaling molecule with a variety of effects. For example, it can inhibit the protease SENP1, which leads to the remodeling of the anaphase-promoting complex (APC/C). This process stimulates the timely degradation of cell cycle cyclins, controlling the cell cycle and proliferation (Liu et al., 2023). Additionally, lactate can be attached to various proteins in a process known as lactylation. This post-translational modification of proteins can regulate gene expression (Zhang et al., 2019), cellular metabolism (Chen et al., 2024), and central immune responses (Sanmarco et al., 2023). Beyond its direct regulatory functions, the signaling role of lactate also depends on the activation of the GPR81. In the periphery, when lactate binds to GPR81, it can inhibit lipolysis (Sun et al., 2016), regulate osteoblast differentiation (Wu et al., 2018), and stimulate an increase in the diameter of muscle fibers (Ohno et al., 2018). Activation of GPR81 in the CNS stimulates angiogenesis (Chaudhari et al., 2022) and neurogenesis (Lambertus et al., 2021), regulates the hippocampal mitochondrial quality control system (Shang et al., 2023) and influences excitability (Herrera-Lopez and Galvan, 2018). Studies show that the central benefits of HIIT, including improved neurogenesis and angiogenesis, are not observed in systemic GPR81 knockout models compared to control groups (Lambertus et al., 2021; Chaudhari et al., 2022). Our research also demonstrates that the knockdown of GPR81 in the hippocampus directly damages synaptic structure and decreases learning and memory functions. Additionally, the enhanced synaptic remodeling seen with HIIT is diminished with GPR81 knockdown. Together with prior reports showing that systemic GPR81 loss-of-function blunts HIIT-related neurogenesis and angiogenesis, our hippocampus-specific knockdown data support a key role of GPR81 in mediating structural synaptic adaptations and memory improvement induced by HIIT. Therefore, HIIT-associated elevations in lactate and hippocampal GPR81 signaling may represent an important contributor to exercise-related memory benefits.

GPCRs can exert their functions through the different subunits of the G proteins to which they are coupled (Kankanamge et al., 2022; McIntire, 2022). As a Gi-type GPCR, GPR81 can inhibit AC through the Gα subunit upon activation, decreasing cAMP levels (Cai et al., 2008; Ge et al., 2008). This modulation has various implications, including stimulating Malonyl-CoA: ACP transacylase (MAT) activity in adipocytes and the promotion of fat synthesis. Treatment with pertussis toxin, a Gi signaling inhibitor, has been shown to block this effect of GPR81 (Cai et al., 2008). Moreover, AC inhibition reduces PKA activation, potentially suppressing PKA-induced pathways like HIF-1α ubiquitination, which could exacerbate disease progression (Luo et al., 2022).On the other hand, lactate activates the GPR81-Gβγ-PLC-PKC signaling pathway, which regulates osteoblast differentiation through parathyroid hormone (Wu et al., 2018). Additionally, GPR81-Gβγ is involved in the chemotherapy resistance mechanism of cancer cells to doxorubicin, mediated by the ATP-binding cassette sub-family B member 1 (ABCB1) transporter protein. Inhibition of the PKC pathway suppresses the GPR81-mediated upregulation of ABCB1 function, resembling the function of Gα (Wagner et al., 2017). However, the specific mechanisms of GPR81 in the CNS, particularly its role in hippocampal synaptic plasticity, are still poorly understood. Our study examines whether GPR81 operates in the CNS similar to its peripheral functions, involving two pathways. *In vivo* research shows that HIIT boosts PLC levels and reduces AC protein expression; the knockdown of GPR81 reverses these effects. *In vitro* experiments investigate the impact of GPR81 agonists, AC agonists, and PLC inhibitors. The results reveal that GPR81 activation decreases AC expression, increases PLC expression, and enhances proteins linked to synaptic plasticity. However, combining these agents suppresses the GPR81-mediated promotion of synaptic plasticity-related protein expression. These results support a GPR81-dependent dual-branch signaling framework that may contribute to HIIT-induced hippocampal synaptic remodeling *via* convergence on ERK1/2.

Synaptic plasticity is an intricate process that involves the interaction of numerous proteins. Research has demonstrated that proteins associated with synaptic plasticity can be controlled by the ERK1/2-MSK1/P90RSK-CREB pathway (Sgambato et al., 1998; Tao et al., 1998; Davis et al., 2000; Hunter et al., 2017; Balu and Coyle, 2018; Chen et al., 2018). Physical exercise has been shown to elevate the phosphorylation of ERK1/2, leading to improved brain function (Shen et al., 2001; Guo et al., 2008; Zhang et al., 2014; Li et al., 2017). Additionally, various studies suggest that alterations in ERK1/2 can be influenced by signals from different GPCRs, such as Gi-type GPCRs able to enhance ERK1/2 expression through multiple pathways (May and Hill, 2008). We conducted *in vitro* pharmacological experiments to examine whether GPR81 activates ERK1/2 through the downstream dual subunit pathway, ultimately enhancing synaptic plasticity. The results showed that inhibiting ERK1/2 decreased the promoting effect after GPR81 activation, indicating that GPR81 influences hippocampal synaptic plasticity *via* the ERK1/2 pathway. These synaptic plasticity-related proteins have distinct roles in regulating synaptic function. For instance, neurotrophic factors like BDNF activate their receptors, such as TrkB, to influence postsynaptic signaling pathways, thereby regulating protein synthesis and synaptic function (Schratt et al., 2004; Leal et al., 2014). Other key proteins, like SYN and PSD95, play critical roles in maintaining and regulating synaptic architecture (Baldelli et al., 2007; Xu, 2011). Additionally, proteins like NMDAR modulate synaptic transmission efficiency and impact synaptic plasticity phenomena such as long-term potentiation and depression (Krapivinsky et al., 2003; Yoshii and Constantine-Paton, 2007). Immediate early genes like ARC, Zif268, and c-Fos can regulate gene expression patterns in neurons, thereby influencing the structure and function of synapses (He et al., 2002; McDade et al., 2009; Shepherd and Bear, 2011).

Overall, our data provide relatively convergent support for the lactate/GPR81-ERK1/2 signaling axis and a subset of proteins related to synaptic remodeling. Nevertheless, several aspects could be further strengthened, including the translational interpretability of the *in vitro* model, the consistency of specific readouts across experimental systems, and the degree to which structural, molecular, and behavioral evidence can be integrated into a unified chain of inference. First, for mechanistic interrogation *in vitro*, we employed N2a cells instead of primary hippocampal neurons. Although N2a cells cannot fully recapitulate the mature state of hippocampal neurons or their multicellular microenvironment, they can still provide informative supportive evidence at the mechanistic level. Prior studies have also utilized N2a cells as a cellular model to investigate GPR81 signaling (Shen et al., 2015). In our previous work on lactate/GPR81-related mitochondrial mechanisms, key conclusions were consistent across validations performed in both N2a cells (Shang et al., 2023) and primary hippocampal neurons (Hu et al., 2021), further supporting the utility of N2a cells as a reference system for hippocampus-relevant mechanistic probing.

Notably, N2a cells exhibit pronounced condition dependence and heterogeneity in NMDAR-related phenotypes. Some reports indicate that certain N2a clones lack detectable NMDAR expression and glutamate responsiveness (LePage et al., 2005), while others have identified NR2B protein expression under differentiation conditions (Wang et al., 2004) and observed NMDA/MK-801-sensitive Ca^2+^ responses (Dar et al., 2017; Dar et al., 2018). Together with the clear NMDAR band observed in our Western blotting (Figure 8A), these findings suggest that NMDAR-related outcomes are strongly influenced by clone selection and culture conditions, which may reasonably account for the incomplete concordance of NMDAR changes between *in vivo* and *in vitro* experiments. Additionally, activity-dependent transcription factors display marked temporal and contextual sensitivity. Immediate early genes (IEGs) such as c-Fos, Egr1/Zif268, and Arc are rapidly induced within approximately 30–90 min after neuronal activation and then decay quickly (Lituma et al., 2022; Das et al., 2023; Bulthuis et al., 2025), with kinetics shaped by stimulus intensity (Tyssowski et al., 2018), cellular maturity (Stroud et al., 2020), and input specificity (Lituma et al., 2022). Moreover, Arc and related IEGs can exhibit multi-wave dynamics and region-dependent heterogeneity (Das et al., 2023; Bulthuis et al., 2025), and exercise-evoked peaks can be transient (Cheng et al., 2025). Accordingly, a fixed sampling window may underestimate the dynamic range and true temporal ordering of these transcriptional events. Taken together, N2a-derived readouts (including NMDAR and IEG changes) should be interpreted as context-dependent mechanistic support for the GPR81-ERK1/2-CREB signaling framework, rather than as a complete *in vitro* recapitulation of *in vivo* synaptic plasticity mechanisms.

Second, the streams of structural, molecular, and behavioral evidence were not fully aligned at the level of hippocampal subregion sampling and the corresponding functional phenotype, which may limit the precision of cross-modality interpretation. Specifically, TEM analyses primarily focused on the CA1 region, whereas Golgi staining and Sholl analyses centered on DG neurons. In contrast, Western blotting and qPCR relied on whole-hippocampus homogenates, potentially diluting subregion-specific signals. Furthermore, the Y-maze is not considered a gold-standard assay for learning and memory and may not comprehensively capture the multidimensional features of spatial learning and memory. Future research could strengthen inferences by implementing subregion-resolved analyses that align structural and molecular readouts within the same hippocampal subfield, and by incorporating more targeted behavioral paradigms (e.g., the Morris water maze or Barnes maze) alongside subregion-specific electrophysiological assessments (e.g., synaptic currents and long-term potentiation/depression) to more directly map signaling alterations onto functional synaptic plasticity.

## 5 Conclusion

In conclusion, our findings indicate that lactate/GPR81 signaling is required for key components of HIIT-induced hippocampal synaptic remodeling and memory improvement. *In vivo* knockdown experiments support the necessity of hippocampal GPR81 for the structural and cognitive benefits of HIIT, while *in vitro* pharmacological results provide mechanistic support for a dual-branch GPR81-to-ERK1/2 working model involving the Gα-AC-RAP1 and Gβγ-PLC-PKC pathways (Graphical abstract). These results highlight the potential relevance of HIIT-associated lactate signaling to the design of exercise-based approaches for cognitive enhancement.

## Data Availability

The datasets presented in this study can be found in online repositories. The names of the repository/repositories and accession number(s) can be found in the article/Supplementary Material.

## References

[B1] Afzalpour M. E. Chadorneshin H. T. Foadoddini M. Eivari H. A. (2015). Comparing interval and continuous exercise training regimens on neurotrophic factors in rat brain. Physiol. Behav. 147, 78–83. 10.1016/j.physbeh.2015.04.012 25868740

[B2] Baldelli P. Fassio A. Valtorta F. Benfenati F. (2007). Lack of synapsin I reduces the readily releasable pool of synaptic vesicles at central inhibitory synapses. J. Neurosci. 27 (49), 13520–13531. 10.1523/JNEUROSCI.3151-07.2007 18057210 PMC6673103

[B3] Balu D. T. Coyle J. T. (2018). Altered CREB binding to activity-dependent genes in serine racemase deficient mice, a mouse model of schizophrenia. ACS Chem. Neurosci. 9 (9), 2205–2209. 10.1021/acschemneuro.7b00404 29172439 PMC5971149

[B4] Banasr M. Sanacora G. Esterlis I. (2021). Macro- and microscale stress-associated alterations in brain structure: translational link with depression. Biol. Psychiatry 90 (2), 118–127. 10.1016/j.biopsych.2021.04.004 34001371

[B5] Beneke R. Leithauser R. M. Ochentel O. (2011). Blood lactate diagnostics in exercise testing and training. Int. J. Sports Physiol. Perform. 6 (1), 8–24. 10.1123/ijspp.6.1.8 21487146

[B6] Bittar P. G. Charnay Y. Pellerin L. Bouras C. Magistretti P. J. (1996). Selective distribution of lactate dehydrogenase isoenzymes in neurons and astrocytes of human brain. J. Cereb. Blood Flow. Metab. 16 (6), 1079–1089. 10.1097/00004647-199611000-00001 8898679

[B7] Bonvento G. Bolanos J. P. (2021). Astrocyte-neuron metabolic cooperation shapes brain activity. Cell Metab. 33 (8), 1546–1564. 10.1016/j.cmet.2021.07.006 34348099

[B8] Brooks G. A. (2018). The science and translation of lactate shuttle theory. Cell Metab. 27 (4), 757–785. 10.1016/j.cmet.2018.03.008 29617642

[B9] Brooks G. A. (2020). Lactate as a fulcrum of metabolism. Redox Biol. 35, 101454. 10.1016/j.redox.2020.101454 32113910 PMC7284908

[B10] Brooks G. A. Osmond A. D. Arevalo J. A. Curl C. C. Duong J. J. Horning M. A. (2022). Lactate as a major myokine and exerkine. Nat. Rev. Endocrinol. 18 (11), 712. 10.1038/s41574-022-00724-0 35915256

[B11] Bulthuis N. E. Quintana L. I. Stackmann M. Denny C. A. (2025). Immediate-early genes Arc and c-Fos show divergent brain-wide expression following contextual fear conditioning. Commun. Biol. 8 (1), 1452. 10.1038/s42003-025-08856-5 41068278 PMC12511597

[B12] Cai T. Q. Ren N. Jin L. Cheng K. Kash S. Chen R. (2008). Role of GPR81 in lactate-mediated reduction of adipose lipolysis. Biochem. Biophys. Res. Commun. 377 (3), 987–991. 10.1016/j.bbrc.2008.10.088 18952058

[B13] Calverley T. A. Ogoh S. Marley C. J. Steggall M. Marchi N. Brassard P. (2020). HIITing the brain with exercise: mechanisms, consequences and practical recommendations. J. Physiol. 598 (13), 2513–2530. 10.1113/JP275021 32347544

[B14] Chaudhari P. Madaan A. Rivera J. C. Charfi I. Habelrih T. Hou X. (2022). Neuronal GPR81 regulates developmental brain angiogenesis and promotes brain recovery after a hypoxic ischemic insult. J. Cereb. Blood Flow. Metab. 42 (7), 1294–1308. 10.1177/0271678X221077499 35107038 PMC9207492

[B15] Chen J. Niu Q. Xia T. Zhou G. Li P. Zhao Q. (2018). ERK1/2-mediated disruption of BDNF-TrkB signaling causes synaptic impairment contributing to fluoride-induced developmental neurotoxicity. Toxicology 410, 222–230. 10.1016/j.tox.2018.08.009 30130557

[B16] Chen Y. Wu J. Zhai L. Zhang T. Yin H. Gao H. (2024). Metabolic regulation of homologous recombination repair by MRE11 lactylation. Cell 187 (2), 294–311 e221. 10.1016/j.cell.2023.11.022 38128537 PMC11725302

[B17] Cheng T. Douglas Affonso F. Yu J. Zhong Y. Ma Z. Hussain A. (2025). Rapid antidepressant effect of single-bout exercise is mediated by adiponectin-induced APPL1 nucleus translocation in anterior cingulate cortex. Mol. Psychiatry 30 (12), 5760–5776. 10.1038/s41380-025-03317-1 41139155 PMC12602346

[B18] Choi D. I. Kaang B. K. (2022). Interrogating structural plasticity among synaptic engrams. Curr. Opin. Neurobiol. 75, 102552. 10.1016/j.conb.2022.102552 35598549

[B19] Constans A. Pin-Barre C. Molinari F. Temprado J. J. Brioche T. Pellegrino C. (2021). High-intensity interval training is superior to moderate intensity training on aerobic capacity in rats: impact on hippocampal plasticity markers. Behav. Brain Res. 398, 112977. 10.1016/j.bbr.2020.112977 33141075

[B20] Dachtler J. Hardingham N. R. Glazewski S. Wright N. F. Blain E. J. Fox K. (2011). Experience-dependent plasticity acts via GluR1 and a novel neuronal nitric oxide synthase-dependent synaptic mechanism in adult cortex. J. Neurosci. 31 (31), 11220–11230. 10.1523/JNEUROSCI.1590-11.2011 21813683 PMC3508401

[B21] Dar N. J. Bhat J. A. Satti N. K. Sharma P. R. Hamid A. Ahmad M. (2017). Withanone, an active constituent from Withania somnifera, affords protection against NMDA-induced excitotoxicity in neuron-like cells. Mol. Neurobiol. 54 (7), 5061–5073. 10.1007/s12035-016-0044-7 27541286

[B22] Dar N. J. Satti N. K. Dutt P. Hamid A. Ahmad M. (2018). Attenuation of glutamate-induced excitotoxicity by Withanolide-A in neuron-like cells: role for PI3K/Akt/MAPK signaling pathway. Mol. Neurobiol. 55 (4), 2725–2739. 10.1007/s12035-017-0515-5 28447311

[B23] Das S. Lituma P. J. Castillo P. E. Singer R. H. (2023). Maintenance of a short-lived protein required for long-term memory involves cycles of transcription and local translation. Neuron 111 (13), 2051–2064 e2056. 10.1016/j.neuron.2023.04.005 37100055 PMC10330212

[B24] Davis S. Vanhoutte P. Pages C. Caboche J. Laroche S. (2000). The MAPK/ERK cascade targets both Elk-1 and cAMP response element-binding protein to control long-term potentiation-dependent gene expression in the dentate gyrus in vivo. J. Neurosci. 20 (12), 4563–4572. 10.1523/JNEUROSCI.20-12-04563.2000 10844026 PMC6772466

[B25] Dos Santos J. R. Bortolanza M. Ferrari G. D. Lanfredi G. P. do Nascimento G. C. Azzolini A. (2020). One-week high-intensity interval training increases hippocampal plasticity and mitochondrial content without changes in redox state. Antioxidants (Basel) 9 (5), 445. 10.3390/antiox9050445 32455608 PMC7278594

[B26] El Hayek L. Khalifeh M. Zibara V. Abi Assaad R. Emmanuel N. Karnib N. (2019). Lactate mediates the effects of exercise on learning and memory through SIRT1-Dependent activation of hippocampal brain-derived neurotrophic factor (BDNF). J. Neurosci. 39 (13), 2369–2382. 10.1523/JNEUROSCI.1661-18.2019 30692222 PMC6435829

[B27] Erickson K. I. Hillman C. Stillman C. M. Ballard R. M. Bloodgood B. Conroy D. E. (2019). Physical activity, cognition, and brain outcomes: a review of the 2018 physical activity guidelines. Med. Sci. Sports Exerc 51 (6), 1242–1251. 10.1249/MSS.0000000000001936 31095081 PMC6527141

[B28] Ge H. Weiszmann J. Reagan J. D. Gupte J. Baribault H. Gyuris T. (2008). Elucidation of signaling and functional activities of an orphan GPCR, GPR81. J. Lipid Res. 49 (4), 797–803. 10.1194/jlr.M700513-JLR200 18174606

[B29] Giachello C. N. Fiumara F. Giacomini C. Corradi A. Milanese C. Ghirardi M. (2010). MAPK/Erk-dependent phosphorylation of synapsin mediates formation of functional synapses and short-term homosynaptic plasticity. J. Cell Sci. 123 (Pt 6), 881–893. 10.1242/jcs.056846 20159961

[B30] Guo M. Lin V. Davis W. Huang T. Carranza A. Sprague S. (2008). Preischemic induction of TNF-alpha by physical exercise reduces blood-brain barrier dysfunction in stroke. J. Cereb. Blood Flow. Metab. 28 (8), 1422–1430. 10.1038/jcbfm.2008.29 18414498

[B31] Guo G. Kang L. Geng D. Han S. Li S. Du J. (2020). Testosterone modulates structural synaptic plasticity of primary cultured hippocampal neurons through ERK - CREB signalling pathways. Mol. Cell Endocrinol. 503, 110671. 10.1016/j.mce.2019.110671 31805308

[B32] He J. Yamada K. Nabeshima T. (2002). A role of Fos expression in the CA3 region of the hippocampus in spatial memory formation in rats. Neuropsychopharmacology 26 (2), 259–268. 10.1016/S0893-133X(01)00332-3 11790521

[B33] Herrera-Lopez G. Galvan E. J. (2018). Modulation of hippocampal excitability via the hydroxycarboxylic acid receptor 1. Hippocampus 28 (8), 557–567. 10.1002/hipo.22958 29704292

[B34] Herrera-Lopez G. Griego E. Galvan E. J. (2020). Lactate induces synapse-specific potentiation on CA3 pyramidal cells of rat hippocampus. PLoS One 15 (11), e0242309. 10.1371/journal.pone.0242309 33180836 PMC7660554

[B35] Hu J. Cai M. Shang Q. Li Z. Feng Y. Liu B. (2021). Elevated lactate by high-intensity interval training regulates the hippocampal BDNF expression and the mitochondrial quality control system. Front. Physiol. 12, 629914. 10.3389/fphys.2021.629914 33716776 PMC7946986

[B36] Hunter C. J. Remenyi J. Correa S. A. Privitera L. Reyskens K. Martin K. J. (2017). MSK1 regulates transcriptional induction of Arc/Arg3.1 in response to neurotrophins. FEBS Open Bio 7 (6), 821–834. 10.1002/2211-5463.12232 28593137 PMC5458472

[B37] Jacob N. So I. Sharma B. Marzolini S. Tartaglia M. C. Oh P. (2023). Effects of high-intensity interval training protocols on blood lactate levels and cognition in healthy adults: systematic review and meta-regression. Sports Med. 53 (5), 977–991. 10.1007/s40279-023-01815-2 36917435

[B38] Kankanamge D. Tennakoon M. Karunarathne A. Gautam N. (2022). G protein gamma subunit, a hidden master regulator of GPCR signaling. J. Biol. Chem. 298 (12), 102618. 10.1016/j.jbc.2022.102618 36272647 PMC9678972

[B39] Kim J. J. Diamond D. M. (2002). The stressed hippocampus, synaptic plasticity and lost memories. Nat. Rev. Neurosci. 3 (6), 453–462. 10.1038/nrn849 12042880

[B40] Krapivinsky G. Krapivinsky L. Manasian Y. Ivanov A. Tyzio R. Pellegrino C. (2003). The NMDA receptor is coupled to the ERK pathway by a direct interaction between NR2B and RasGRF1. Neuron 40 (4), 775–784. 10.1016/s0896-6273(03)00645-7 14622581

[B41] Lambertus M. Overberg L. T. Andersson K. A. Hjelden M. S. Hadzic A. Haugen O. P. (2021). L-lactate induces neurogenesis in the mouse ventricular-subventricular zone via the lactate receptor HCA(1). Acta Physiol. (Oxf) 231 (3), e13587. 10.1111/apha.13587 33244894

[B42] Lauritzen K. H. Morland C. Puchades M. Holm-Hansen S. Hagelin E. M. Lauritzen F. (2014). Lactate receptor sites link neurotransmission, neurovascular coupling, and brain energy metabolism. Cereb. Cortex 24 (10), 2784–2795. 10.1093/cercor/bht136 23696276

[B43] Leal G. Comprido D. Duarte C. B. (2014). BDNF-induced local protein synthesis and synaptic plasticity. *Neuropharmacology* 76 Pt C, 639–656. 10.1016/j.neuropharm.2013.04.005 23602987

[B44] Lei Z. Mozaffaritabar S. Kawamura T. Koike A. Kolonics A. Keringer J. (2024). The effects of long-term lactate and high-intensity interval training (HIIT) on brain neuroplasticity of aged mice. Heliyon 10 (2), e24421. 10.1016/j.heliyon.2024.e24421 38293399 PMC10826720

[B45] LePage K. T. Dickey R. W. Gerwick W. H. Jester E. L. Murray T. F. (2005). On the use of neuro-2a neuroblastoma cells versus intact neurons in primary culture for neurotoxicity studies. Crit. Rev. Neurobiol. 17 (1), 27–50. 10.1615/critrevneurobiol.v17.i1.20 16307526

[B46] Li D. J. Li Y. H. Yuan H. B. Qu L. F. Wang P. (2017). The novel exercise-induced hormone irisin protects against neuronal injury via activation of the Akt and ERK1/2 signaling pathways and contributes to the neuroprotection of physical exercise in cerebral ischemia. Metabolism 68, 31–42. 10.1016/j.metabol.2016.12.003 28183451

[B47] Lituma P. J. Singer R. H. Das S. Castillo P. E. (2022). Real-time imaging of Arc/Arg3.1 transcription *ex vivo* reveals input-specific immediate early gene dynamics. Proc. Natl. Acad. Sci. U. S. A. 119 (38), e2123373119. 10.1073/pnas.2123373119 36095210 PMC9499544

[B48] Liu W. Wang Y. Bozi L. H. M. Fischer P. D. Jedrychowski M. P. Xiao H. (2023). Lactate regulates cell cycle by remodelling the anaphase promoting complex. Nature 616 (7958), 790–797. 10.1038/s41586-023-05939-3 36921622 PMC12175651

[B49] Luo M. Zhu J. Ren J. Tong Y. Wang L. Ma S. (2022). Lactate increases tumor malignancy by promoting tumor small extracellular vesicles production via the GPR81-cAMP-PKA-HIF-1alpha axis. Front. Oncol. 12, 1036543. 10.3389/fonc.2022.1036543 36531060 PMC9753130

[B50] Magistretti P. J. Allaman I. (2018). Lactate in the brain: from metabolic end-product to signalling molecule. Nat. Rev. Neurosci. 19 (4), 235–249. 10.1038/nrn.2018.19 29515192

[B51] Maharana C. Sharma K. P. Sharma S. K. (2013). Feedback mechanism in depolarization-induced sustained activation of extracellular signal-regulated kinase in the hippocampus. Sci. Rep. 3, 1103. 10.1038/srep01103 23346360 PMC3551232

[B52] May L. T. Hill S. J. (2008). ERK phosphorylation: spatial and temporal regulation by G protein-coupled receptors. Int. J. Biochem. Cell Biol. 40 (10), 2013–2017. 10.1016/j.biocel.2008.04.001 18502166

[B53] McDade D. M. Conway A. M. James A. B. Morris B. J. (2009). Activity-dependent gene transcription as a long-term influence on receptor signalling. Biochem. Soc. Trans. 37 (Pt 6), 1375–1377. 10.1042/BST0371375 19909279

[B54] McIntire W. E. (2022). A model for how Gbetagamma couples Galpha to GPCR. J. Gen. Physiol. 154 (5), e202112982. 10.1085/jgp.202112982 35333292 PMC8961292

[B55] Miningou N. Blackwell K. T. (2020). The road to ERK activation: do neurons take alternate routes? Cell Signal 68, 109541. 10.1016/j.cellsig.2020.109541 31945453 PMC7127974

[B56] Morland C. Lauritzen K. H. Puchades M. Holm-Hansen S. Andersson K. Gjedde A. (2015). The lactate receptor, G-protein-coupled receptor 81/hydroxycarboxylic acid receptor 1: expression and action in brain. J. Neurosci. Res. 93 (7), 1045–1055. 10.1002/jnr.23593 25881750

[B57] Morland C. Andersson K. A. Haugen O. P. Hadzic A. Kleppa L. Gille A. (2017). Exercise induces cerebral VEGF and angiogenesis via the lactate receptor HCAR1. Nat. Commun. 8, 15557. 10.1038/ncomms15557 28534495 PMC5457513

[B58] Muller P. Duderstadt Y. Lessmann V. Muller N. G. (2020). Lactate and BDNF: key mediators of exercise induced neuroplasticity? J. Clin. Med. 9 (4), 1136. 10.3390/jcm9041136 32326586 PMC7230639

[B59] Neves G. Cooke S. F. Bliss T. V. (2008). Synaptic plasticity, memory and the hippocampus: a neural network approach to causality. Nat. Rev. Neurosci. 9 (1), 65–75. 10.1038/nrn2303 18094707

[B60] Ohno Y. Oyama A. Kaneko H. Egawa T. Yokoyama S. Sugiura T. (2018). Lactate increases myotube diameter via activation of MEK/ERK pathway in C2C12 cells. Acta Physiol. (Oxf) 223 (2), e13042. 10.1111/apha.13042 29377587

[B61] Okamoto M. Mizuuchi D. Omura K. Lee M. Oharazawa A. Yook J. S. (2021). High-intensity intermittent training enhances spatial memory and hippocampal neurogenesis associated with BDNF signaling in rats. Cereb. Cortex 31 (9), 4386–4397. 10.1093/cercor/bhab093 33982757

[B62] Pierre K. Pellerin L. (2005). Monocarboxylate transporters in the central nervous system: distribution, regulation and function. J. Neurochem. 94 (1), 1–14. 10.1111/j.1471-4159.2005.03168.x 15953344

[B63] Ramos J. S. Dalleck L. C. Tjonna A. E. Beetham K. S. Coombes J. S. (2015). The impact of high-intensity interval training versus moderate-intensity continuous training on vascular function: a systematic review and meta-analysis. Sports Med. 45 (5), 679–692. 10.1007/s40279-015-0321-z 25771785

[B64] Sanmarco L. M. Rone J. M. Polonio C. M. Fernandez Lahore G. Giovannoni F. Ferrara K. (2023). Lactate limits CNS autoimmunity by stabilizing HIF-1alpha in dendritic cells. Nature 620 (7975), 881–889. 10.1038/s41586-023-06409-6 37558878 PMC10725186

[B65] Schratt G. M. Nigh E. A. Chen W. G. Hu L. Greenberg M. E. (2004). BDNF regulates the translation of a select group of mRNAs by a mammalian target of rapamycin-phosphatidylinositol 3-kinase-dependent pathway during neuronal development. J. Neurosci. 24 (33), 7366–7377. 10.1523/JNEUROSCI.1739-04.2004 15317862 PMC6729778

[B66] Sgambato V. Pages C. Rogard M. Besson M. J. Caboche J. (1998). Extracellular signal-regulated kinase (ERK) controls immediate early gene induction on corticostriatal stimulation. J. Neurosci. 18 (21), 8814–8825. 10.1523/JNEUROSCI.18-21-08814.1998 9786988 PMC6793547

[B67] Shang Q. Bian X. Zhu L. Liu J. Wu M. Lou S. (2023). Lactate mediates high-intensity interval training-induced promotion of hippocampal mitochondrial function through the GPR81-ERK1/2 pathway. Antioxidants (Basel) 12 (12), 2087. 10.3390/antiox12122087 38136207 PMC10740508

[B68] Shen H. Tong L. Balazs R. Cotman C. W. (2001). Physical activity elicits sustained activation of the cyclic AMP response element-binding protein and mitogen-activated protein kinase in the rat hippocampus. Neuroscience 107 (2), 219–229. 10.1016/s0306-4522(01)00315-3 11731096

[B69] Shen Z. Jiang L. Yuan Y. Deng T. Zheng Y. R. Zhao Y. Y. (2015). Inhibition of G protein-coupled receptor 81 (GPR81) protects against ischemic brain injury. CNS Neurosci. Ther. 21 (3), 271–279. 10.1111/cns.12362 25495836 PMC6495224

[B70] Shepherd J. D. Bear M. F. (2011). New views of Arc, a master regulator of synaptic plasticity. Nat. Neurosci. 14 (3), 279–284. 10.1038/nn.2708 21278731 PMC8040377

[B71] Smith K. J. Ainslie P. N. (2017). Regulation of cerebral blood flow and metabolism during exercise. Exp. Physiol. 102 (11), 1356–1371. 10.1113/EP086249 28786150

[B72] Stroud H. Yang M. G. Tsitohay Y. N. Davis C. P. Sherman M. A. Hrvatin S. (2020). An activity-mediated transition in transcription in early postnatal neurons. Neuron 107 (5), 874–890 e878. 10.1016/j.neuron.2020.06.008 32589877 PMC7486250

[B73] Sun J. Ye X. Xie M. Ye J. (2016). Induction of triglyceride accumulation and mitochondrial maintenance in muscle cells by lactate. Sci. Rep. 6, 33732. 10.1038/srep33732 27645401 PMC5028732

[B74] Sun C. Nold A. Fusco C. M. Rangaraju V. Tchumatchenko T. Heilemann M. (2021). The prevalence and specificity of local protein synthesis during neuronal synaptic plasticity. Sci. Adv. 7 (38), eabj0790. 10.1126/sciadv.abj0790 34533986 PMC8448450

[B75] Sweatt J. D. (2001). The neuronal MAP kinase cascade: a biochemical signal integration system subserving synaptic plasticity and memory. J. Neurochem. 76 (1), 1–10. 10.1046/j.1471-4159.2001.00054.x 11145972

[B76] Tao X. Finkbeiner S. Arnold D. B. Shaywitz A. J. Greenberg M. E. (1998). Ca2+ influx regulates BDNF transcription by a CREB family transcription factor-dependent mechanism. Neuron 20 (4), 709–726. 10.1016/s0896-6273(00)81010-7 9581763

[B77] Tyssowski K. M. DeStefino N. R. Cho J. H. Dunn C. J. Poston R. G. Carty C. E. (2018). Different neuronal activity patterns induce different gene expression programs. Neuron 98 (3), 530–546 e511. 10.1016/j.neuron.2018.04.001 29681534 PMC5934296

[B78] VanGuilder H. D. Farley J. A. Yan H. Van Kirk C. A. Mitschelen M. Sonntag W. E. (2011). Hippocampal dysregulation of synaptic plasticity-associated proteins with age-related cognitive decline. Neurobiol. Dis. 43 (1), 201–212. 10.1016/j.nbd.2011.03.012 21440628 PMC3096728

[B79] von Bernhardi R. Bernhardi L. E. Eugenin J. (2017). What is neural plasticity? Adv. Exp. Med. Biol. 1015, 1–15. 10.1007/978-3-319-62817-2_1 29080018

[B80] Wagner W. Kania K. D. Blauz A. Ciszewski W. M. (2017). The lactate receptor (HCAR1/GPR81) contributes to doxorubicin chemoresistance via ABCB1 transporter up-regulation in human cervical cancer HeLa cells. J. Physiol. Pharmacol. 68 (4), 555–564. 29151072

[B81] Wang G. S. Hong C. J. Yen T. Y. Huang H. Y. Ou Y. Huang T. N. (2004). Transcriptional modification by a CASK-interacting nucleosome assembly protein. Neuron 42 (1), 113–128. 10.1016/s0896-6273(04)00139-4 15066269

[B82] Wu F. M. Huang H. G. Hu M. Gao Y. Liu Y. X. (2006). Molecular cloning, tissue distribution and expression in engineered cells of human orphan receptor GPR81. Sheng Wu Gong Cheng Xue Bao 22 (3), 408–412. 10.1016/s1872-2075(06)60036-8 16755919

[B83] Wu Y. Wang M. Zhang K. Li Y. Xu M. Tang S. (2018). Lactate enhanced the effect of parathyroid hormone on osteoblast differentiation via GPR81-PKC-Akt signaling. Biochem. Biophys. Res. Commun. 503 (2), 737–743. 10.1016/j.bbrc.2018.06.069 29913143

[B84] Xu W. (2011). PSD-95-like membrane associated guanylate kinases (PSD-MAGUKs) and synaptic plasticity. Curr. Opin. Neurobiol. 21 (2), 306–312. 10.1016/j.conb.2011.03.001 21450454 PMC3138136

[B85] Yoshii A. Constantine-Paton M. (2007). BDNF induces transport of PSD-95 to dendrites through PI3K-AKT signaling after NMDA receptor activation. Nat. Neurosci. 10 (6), 702–711. 10.1038/nn1903 17515902

[B86] Zhang L. Niu W. He Z. Zhang Q. Wu Y. Jiang C. (2014). Autophagy suppression by exercise pretreatment and p38 inhibition is neuroprotective in cerebral ischemia. Brain Res. 1587, 127–132. 10.1016/j.brainres.2014.08.067 25192645

[B87] Zhang D. Tang Z. Huang H. Zhou G. Cui C. Weng Y. (2019). Metabolic regulation of gene expression by histone lactylation. Nature 574 (7779), 575–580. 10.1038/s41586-019-1678-1 31645732 PMC6818755

[B88] Zhou X. Moon C. Zheng F. Luo Y. Soellner D. Nunez J. L. (2009). N-methyl-D-aspartate-stimulated ERK1/2 signaling and the transcriptional up-regulation of plasticity-related genes are developmentally regulated following in vitro neuronal maturation. J. Neurosci. Res. 87 (12), 2632–2644. 10.1002/jnr.22103 19396876 PMC2857719

