# Supplementary material for: HIIT-induced lactate/GPR81 signaling with dual branches converging on ERK1/2 contributes to hippocampal synaptic remodeling and memory improvement
Source: Front Cell Dev Biol. 2026 Jan 14;14:1699042. doi: 10.3389/fcell.2026.1699042 (PMC12846946; doi:10.3389/fcell.2026.1699042)
Supplement: Supplementary file 1 [file Supplementaryfile1.docx]

Supplementary Material

# Supplementary Figures and Tables

## Supplementary Figures


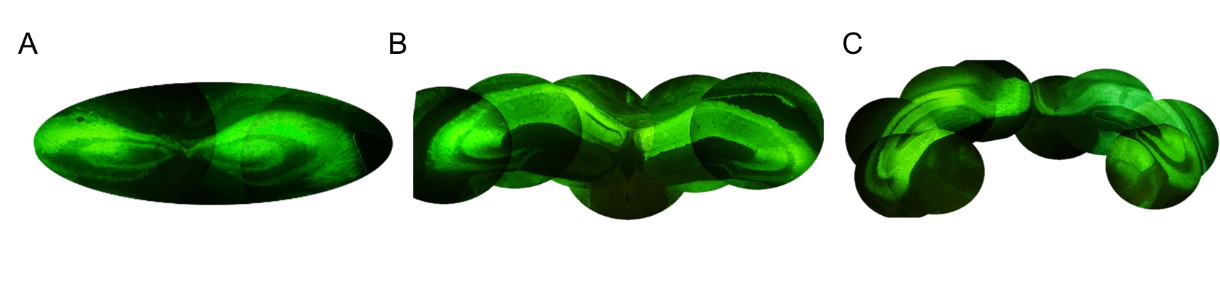


**Supplementary Figure 1.** **Evaluation of viral transduction efficiency for Virus 1 (Hanbio).** Panels A–C represent different hippocampal sections from three individual mice, demonstrating strong EGFP fluorescence, robust hippocampal infection, and minimal non-specific transduction.


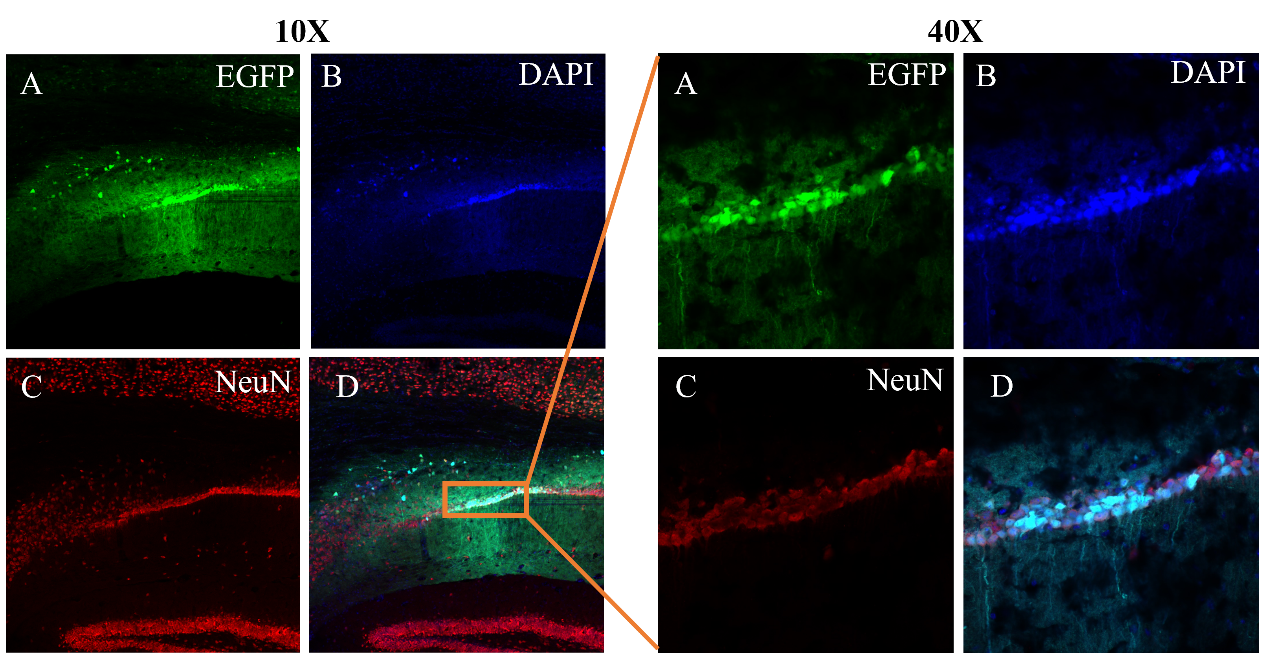


**Supplementary Figure 2.** **Validation of viral transduction efficiency and neuronal specificity in the hippocampus.** (A) EGFP fluorescence (green) indicating viral transduction. (B) DAPI nuclear staining (blue). (C) NeuN immunofluorescence labeling neurons (red). (D) Merged image. The virus showed robust hippocampal transduction with minimal non-specific infection. Based on the results in Figs. 1–2, this vendor’s viral preparation was selected for subsequent in vivo injections.


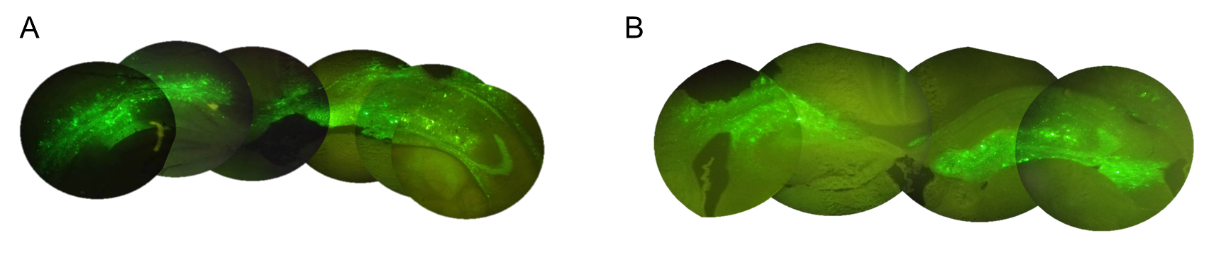


**Supplementary Figure 3.** **Evaluation of viral transduction efficiency for Virus 2 (BrainVTA).** (A–B) Representative hippocampal sections from two individual mice showing EGFP fluorescence (green). The EGFP signal appeared heterogeneous and disorganized, with relatively low hippocampal transduction efficiency and substantial non-specific infection.


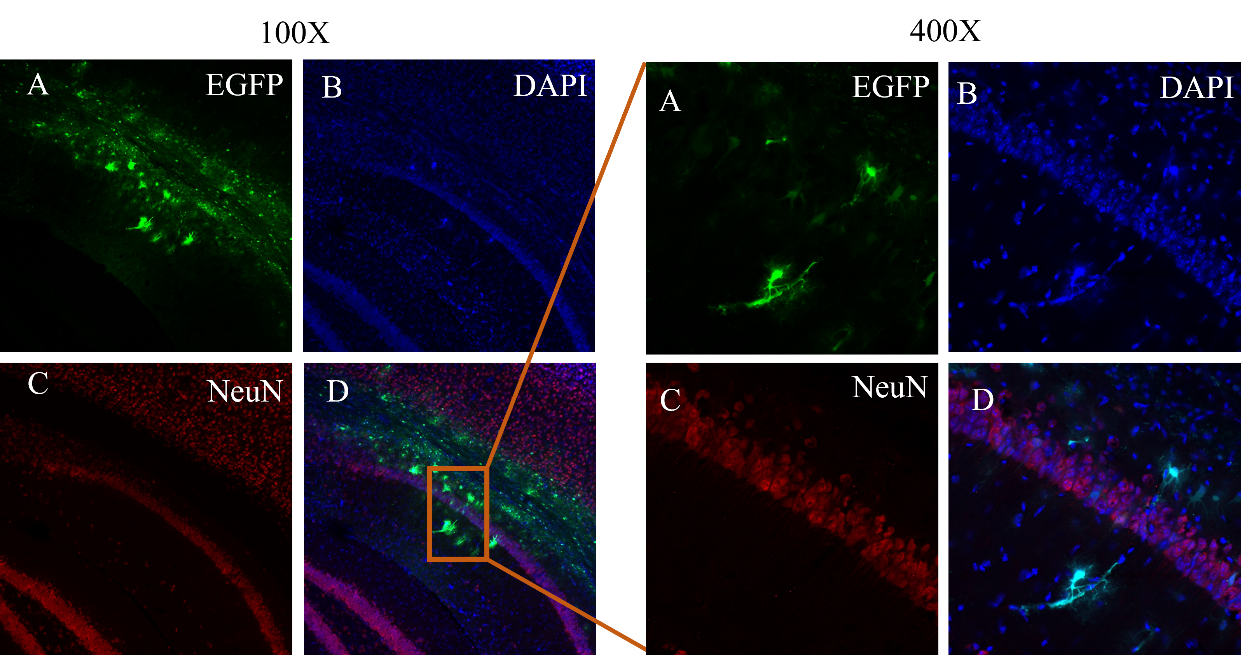


**Supplementary Figure 4. Neuronal specificity of Virus 2 transduction in the hippocampus.** (A) EGFP fluorescence (green) indicating viral transduction. (B) DAPI nuclear staining (blue). (C) NeuN immunofluorescence labeling neurons (red). (D) Merged image. Overall transduction was poor, with low neuronal infection efficiency and disorganized EGFP signals. Consistent with the observations in Fig. 3, this viral preparation was not selected for subsequent experiments.


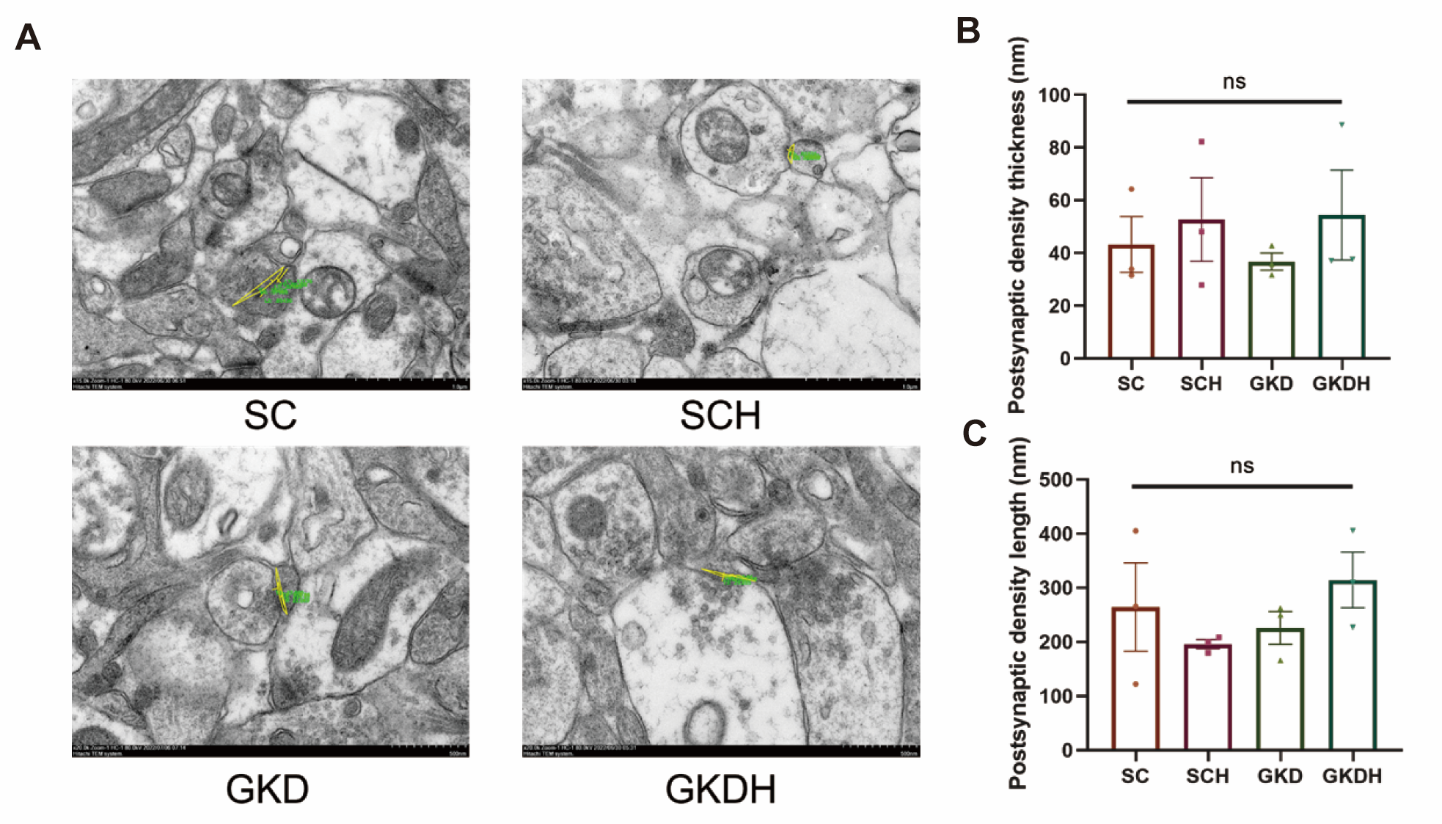


**Supplementary Figure 5.** (A-C) Representative TEM images of postsynaptic density length and thickness from each group, and the corresponding quantitative analysis. The scale bar represents 500 nm. For comparisons among multiple groups, one-way ANOVA was applied. The data are presented as mean ± SD. *n* = 3. ns, no significant difference. SC, scramble control, sedentary; SCH, scramble control + HIIT; GKD, GPR81 knockdown, sedentary; GKDH, GPR81 knockdown + HIIT.


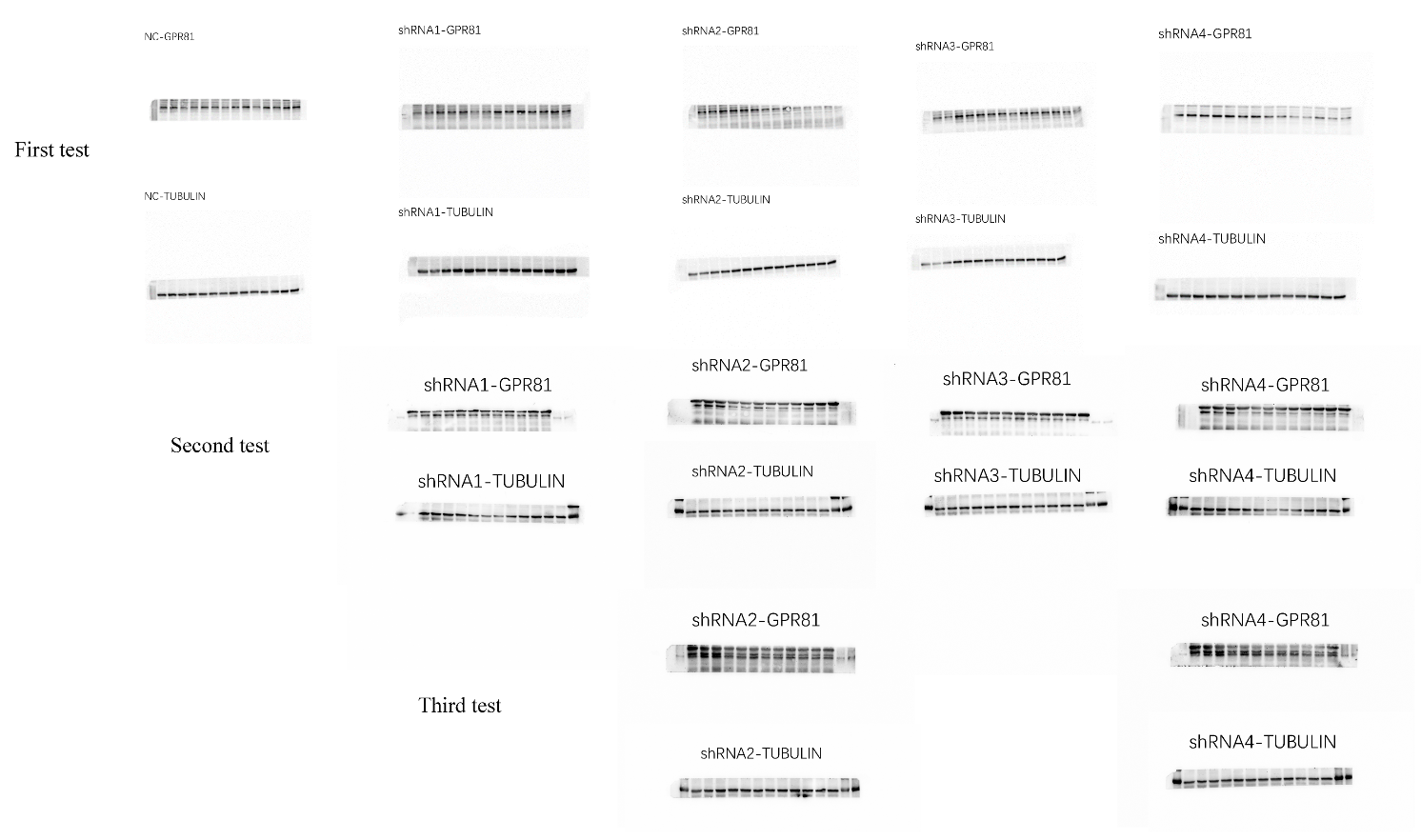


**Supplementary Figure 6.** **The GPR81 shRNA sequence used for the in vivo knockdown experiments was validated more than three times in ex vivo experiments.**
